# Anti-apoptotic HAX-1 suppresses cell apoptosis by promoting c-Abl kinase-involved ROS clearance

**DOI:** 10.1038/s41419-022-04748-2

**Published:** 2022-04-04

**Authors:** Qincai Dong, Dapei Li, Huailong Zhao, Xun Zhang, Yue Liu, Yong Hu, Yi Yao, Lin Zhu, Guang-Fei Wang, Hainan Liu, Ting Gao, Xiayang Niu, Tong Zheng, Caiwei Song, Di Wang, Yu Bai, Jing Jin, Zijing Liu, Yanwen Jin, Ping Li, Cheng Cao, Xuan Liu

**Affiliations:** 1 Beijing Institute of Biotechnology, 27 Taiping Rd, Haidian District, 100850 Beijing, China; 2 Center for Systems Medicine, Institute of Basic Medical Sciences, Chinese Academy of Medical Sciences & Peking Union Medical College, 100005 Beijing, China; 3 Suzhou Institute of Systems Medicine, 215123 Suzhou, China; 4 Jinan Center for Disease Control and Prevention, 250021 Jinan, Shandong China; 5 Institute of Health Sciences, Anhui University, 230601 Hefei, China

**Keywords:** Apoptosis, Kinases

## Abstract

The anti-apoptotic protein HAX-1 has been proposed to modulate mitochondrial membrane potential, calcium signaling and actin remodeling. *HAX-1* mutation or deficiency results in severe congenital neutropenia (SCN), loss of lymphocytes and neurological impairments by largely unknown mechanisms. Here, we demonstrate that the activation of c-Abl kinase in response to oxidative or genotoxic stress is dependent on HAX-1 association. Cellular reactive oxygen species (ROS) accumulation is inhibited by HAX-1-dependent c-Abl activation, which greatly contributes to the antiapoptotic role of HAX-1 in stress. HAX-1 (Q190X), a loss-of-function mutant responsible for SCN, fails to bind with and activate c-Abl, leading to dysregulated cellular ROS levels, damaged mitochondrial membrane potential and eventually apoptosis. The extensive apoptosis of lymphocytes and neurons in *Hax-1*-deficient mice could also be remarkably suppressed by c-Abl activation. These findings underline the important roles of ROS clearance in HAX-1-mediated anti-apoptosis by c-Abl kinase activation, providing new insight into the pathology and treatment of HAX-1-related hereditary disease or tumorigenesis.

## Introduction

The anti-apoptotic protein HAX-1, which was initially identified as HS-1 (hematopoietic lineage cell-specific protein)-associated protein [1], is ubiquitously expressed in various tissues and tumors [2, 3]. Similar to BCL-2 family members, it has BH1- and BH2-like domains and a C-terminal transmembrane domain. HAX-1 is critical for maintaining the inner mitochondrial membrane potential and protecting cells against apoptosis [4–8]. Extensive apoptosis in lymphocytes and neurons [9], even in cardiac myocytes [10] and melanoma cells [11], was observed in *Hax-1-*deficient mice, demonstrating the antiapoptotic role of HAX-1 [1, 12, 13]. Biallelic mutations in the human *HAX-1* gene lead to autosomal recessive severe congenital neutropenia (SCN or Kostmann syndrome) and neurological abnormalities, mainly resulting from the loss of mitochondrial control of apoptosis [14–16]. The mitochondrial proteases Parl and HtrA2 have been reported to participate in HAX-1-induced anti-apoptosis by preventing the accumulation of activated Bax [9, 17]. However, the specific mechanism by which *HAX-1* mutation leads to a variety of physiological aberrations is still unknown.

The nonreceptor tyrosine kinases c-Abl and Arg (abl-related gene, Abl2) are ubiquitously expressed in mammalian tissues with overlapping functions in cell proliferation, apoptosis, adhesion and cell migration [18–20]. The activities of Abl tyrosine kinases were autoinhibited under normal physiological conditions and activated by oxidative or genotoxic stress to facilitate ROS scavenging and DNA repair. c-Abl plays both proapoptotic and antiapoptotic functions depending on the cellular context [21]. While nuclear c-Abl is required for ion irradiation-induced apoptosis by interactions with p53 and p73 [22–24], the cytoplasmic Bcr-Abl kinase and C-terminal truncated forms of c-Abl (loss of nuclear localization signal) are strong inhibitors of apoptosis [25]. *c-Abl*^−/−^ progenitor B cells are more sensitive than wild-type cells to apoptosis induced by growth factor deprivation and glucocorticoid treatment [26]. Importantly, embryos deficient in both c-Abl and Arg exhibit defects in neurulation and die by 11 days postcoitus with massive apoptosis in all tissues [27]. Concordantly, fibroblast cells from *c-Abl*^−/−^*Arg*^−/−^ mice are much more sensitive to ROS stimulus than wild-type cells [28]. ROS activate c-Abl [29–31], either by activating ataxia-telangiectasia mutated (ATM) kinase [32, 33], by facilating PKCδ mediated c-Abl phosphorylation, or activating c-Abl directly. Following the activation by ROS, c-Abl regulates ROS clearance by phosphorylating the key regulators of cellular ROS level such as catalase, glutathione peroxidase, and Prx1 [34].

Our previous work showed that HAX-1 was a candidate association protein of c-Abl by yeast two-hybrid assays. In this study, HAX-1 was determined to be a novel binding partner of c-Abl kinase, and its association with HAX-1 was indispensable for Abl kinase activation induced by oxidative or genotoxic stress. HAX-1-mediated c-Abl activation also partially contributed to HAX-1-mediated anti-apoptosis and provided a clue for understanding HAX-1-related physiological aberrations.

## Results

### HAX-1 interacts with c-Abl kinase in vivo and in vitro

**Fig. 1:**
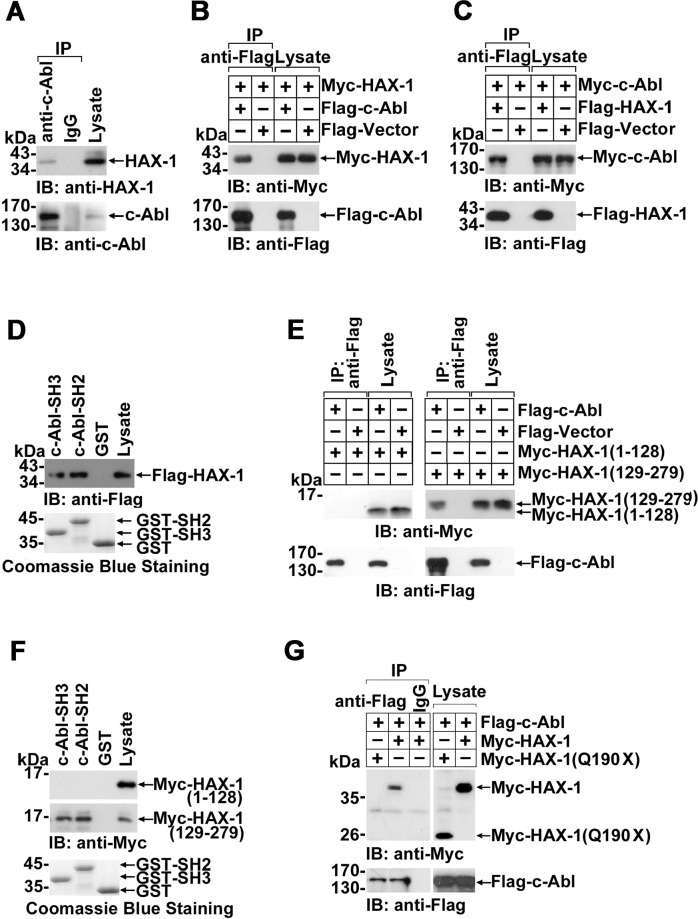
Association of HAX-1 with c-Abl. **A** Lysates from MCF-7 cells were immunoprecipitated with anti-c-Abl antibody and rabbit IgG. The precipitates were fractionated with SDS-PAGE and immunoblotted with anti-HAX-1 and anti-c-Abl antibodies. Whole lysates (2% v/v) were used as controls to confirm HAX-1 and c-Abl expression. **B** HEK 293 cells were co-transfected with Myc-HAX-1 and Flag-c-Abl or Flag-vector as a control. Cell lysates were immunoprecipitated with anti-Flag antibody. The immunoprecipitates were evaluated by SDS-PAGE and immunoblotting and probed with anti-Myc or anti-Flag antibodies. **C** HEK 293 cells were co-transfected with indicated plasmids. To normalize the input level of c-Abl, four-fold amount of Myc-c-Abl expressing plasmid was used in Flag-HAX-1 co-transfection than that in Flag-Vector co-transfection. Cell lysates were analyzed by immunoprecipitation and immunoblotting. **D** Lysates from HEK 293 cells transfected with Flag-HAX-1 were incubated with equal amounts of Sepharose beads conjugated to GST or the GST-c-Abl-SH3/SH2 fusion protein. The absorbates were analyzed by Western blot. Whole lysate (2% v/v) was included as a control. Staining with Coomassie brilliant blue confirmed the presence of GST and the GST-c-Abl-SH3/SH2 fusion protein. **E** Lysates from HEK 293 cells transfected with indicated plasmids were subjected to immunoprecipitation with anti-Flag and SDS-PAGE and subsequently analyzed by immunoblotting. **F** Lysates from HEK 293 cells transfected with indicated plasmids were incubated with GST or GST-c-Abl-SH3 fusion protein. The adsorbates were analyzed by Western blot with anti-Myc antibody. **G** HEK 293 cells were transfected as indicated. To normalize the input level c-Abl, four fold amount of Flag-c-Abl expressing plasmid was used in Myc-HAX-1 co-transfection than that in Myc-HAX-1(Q190X) co-transfection. Lysates were subjected to immunoprecipitation with anti-Flag antibody and subsequently analyzed by Western blot.

To substantiate the potential association of HAX-1 and c-Abl, anti-c-Abl (or IgG as control) immunoprecipitates prepared from MCF-7 cell extracts were subjected to anti-HAX-1 immunoblotting. HAX-1 was detected in anti-c-Abl (but not IgG) immunoprecipitates, indicating the in vivo association of endogenous c-Abl and HAX-1 (Fig. 1A). Furthermore, Flag- or Myc-tagged c-Abl and HAX-1 were exogenously expressed in the cells, and their interactions were also observed by reciprocal immunoprecipitation (Fig. 1B, C). We also noticed that the exogenous Myc-c-Abl level was significantly downregulated by Flag-HAX-1 co-expression (Fig. S1A). In order to obtain a comparable Myc-c-Abl expression level, four fold amount of Myc-c-Abl plasmid was used in Flag-HAX-1 co-transfection than that in Flag-vector co-transfection (Fig. 1C). To define the interaction domain of c-Abl kinase, exogenously expressed Flag-HAX-1 was incubated with GST-c-Abl-SH2-, GST-c-Abl-SH3-, or GST-conjugated Sepharose beads in vitro, and the adsorbates were analyzed by immunoblotting. The results showed that HAX-1 associated to either the SH3 or SH2 domain of c-Abl but not to the GST-only protein (Fig. 1D). No detectable tyrosine phosphorylation was observed in HAX-1 co-expressed with c-Abl, which suggests that c-Abl SH2 domain associates with HAX-1 indirectly (e.g. through c-Abl).

**Fig. 2:**
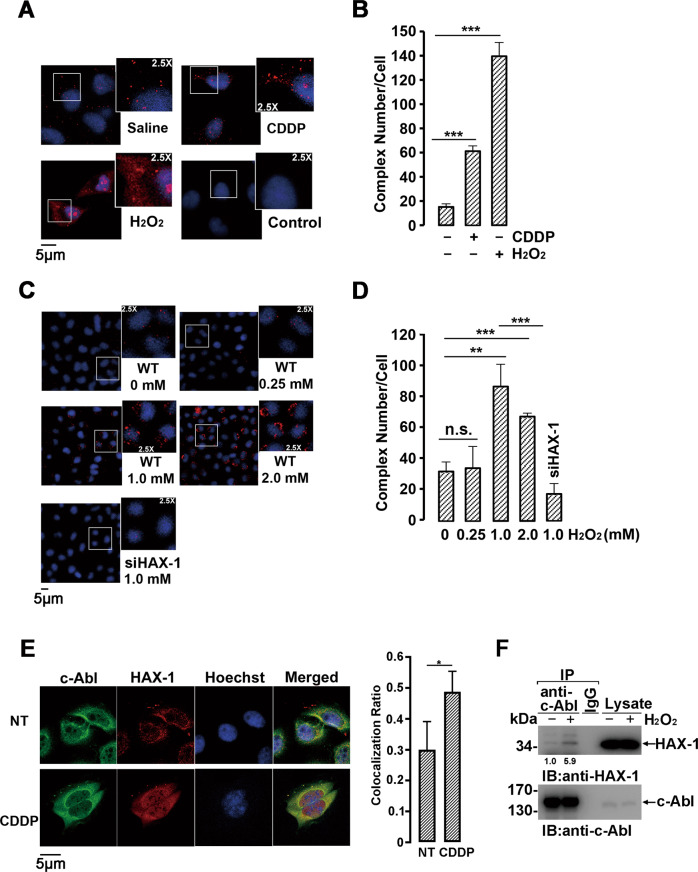
Interaction of c-Abl with HAX-1 in different physiological contexts. **A** An in situ proximity ligation assay (in situ PLA) was used for the detection of HAX-1 and c-Abl binding complexes. MCF-7 cells treated with or without CDDP (25 mM, 8 h) or H_2_O_2_ (1 mM, 3 h) were incubated with target primary antibodies from two different species or with anti-c-Abl antibody as a control. The red spots reveal c-Abl/HAX-1 interaction. Nuclei are stained with Hoechst 33342. Slides were evaluated using an LSM 510 META confocal microscope (Carl Zeiss). Cell images obtained were exported using the Zeiss LSM Image Browser (Carl Zeiss) in TIF format for further analysis. **B** Quantification of HAX-1-c-Abl interaction complexes. The number of complexes per cell was counted in at least three fields. Quantifications were given as the mean±S.D. Representative results are shown from experiments repeated three times. ***p < 0.001, Student’s *t* test. **C** MCF-7 or *HAX-1* siRNA cells treated with the indicated dosage of H_2_O_2_ were subjected to in situ PLA analysis. **D** Quantification of HAX-1-c-Abl interaction complexes. The number of complexes per cell was counted in at least three fields. Quantifications were given as the mean ± S.D. Representative results are shown from experiments repeated three times. n.s., not significant; **p < 0.01, ***p < 0.001, Student’s *t* test. **E** MCF-7 cells treated with or without CDDP (25 mM, 8 h) were incubated with target primary anti-c-Abl and anti-HAX-1 antibodies and then incubated with FITC- or TRITC-linked secondary antibodies. Nuclei are stained with Hoechst 33342 (left). The relative colocalization ratio of HAX-1 with c-Abl was analyzed by ImageJ software (right). At least 15 cells were analyzed, and the data were shown as mean±S.D. *p < 0.05, Student’s *t* test. **F** Lysates prepared from MCF-7 cells treated with or without H_2_O_2_ (1 mM, 3 h) were analyzed by immunoprecipitation and immunoblotting.

The N-terminal of HAX-1 (1–128 a.a.) contains putative but poorly similar Bcl-2 homology domains (BH1 and BH2) and a PEST sequence, and the C-terminus of HAX-1 (129–279 a.a.) contains a conserved C-terminal α-helix domain mainly responsible for interaction with other proteins. Our data showed that only HAX-1 (129–279), but not HAX-1 (1–128), could interact with c-Abl in vivo and in vitro (Fig. 1E, F). In accordance with these findings, HAX-1 (Q190X), a truncated mutant at position 190 leading to neutropenia and neuronal diseases, failed to bind with c-Abl (Fig. 1G). Consistent with Fig. S1A, Flag-c-Abl co-expressed with wild-type HAX-1 demonstrated a significantly decreased protein level (Fig. S1B). To achieve a balanced Flag-c-Abl expression level, 4 fold amount of Flag-c-Abl plasmid was used in wild-type HAX-1 co-transfection than that of HAX-1(Q190X) co-transfection (Fig. 1G). These results implied that the association of c-Abl with the C-terminus of HAX-1 might be involved in the physiological role of HAX-1. Using an in situ proximity ligation assay (in situ PLA), the interaction of endogenous c-Abl and HAX-1 was further confirmed by the observation of fluorescence spots in the cytoplasm (Fig. 2A, upper left panel).

### The association of HAX-1 and c-Abl is strengthened by genotoxic or oxidative stimuli

HAX-1 was reported to be involved in the antagonism of apoptotic processes induced by starvation, cytokine withdrawal, irradiation, and genotoxic or oxidative stresses. We then examined whether the association of HAX-1 and c-Abl was regulated under stress conditions, since c-Abl kinase could also be activated by similar stimuli such as genotoxic drugs, ROS, or ionizing radiation [35, 36]. The intensity of the fluorescence signal in the in situ proximity ligation assay (in situ PLA), was observed to be enhanced (~3-fold) by CDDP treatment (Fig. 2A, B), and more strikingly upregulated (~7-fold) by H_2_O_2_ treatment (Fig. 2A, B) in a dose-dependent manner (Fig. 2C, D). No fluorescence signal of association was observed in *HAX-1* knockdown MCF-7 (MCF-7/HAX-1 RNAi) cells, excluding the existence of false-positive signals in wild-type cells (Fig. 2C, D). In concert with these findings by in situ PLA, it was also noted that a substantial amount of HAX-1 (~50%) colocalized with c-Abl in the cytoplasm after CDDP, compared with less than 30% of HAX-1 occupied by c-Abl under normal physiological conditions (Fig. 2E). Upon H_2_O_2_ stimulation, a significantly reinforced association of HAX-1 with c-Abl was also detected by immunoprecipitation as expected (Fig. 2F and S2A).

### c-Abl kinase is activated by HAX-1 interaction

**Fig. 3:**
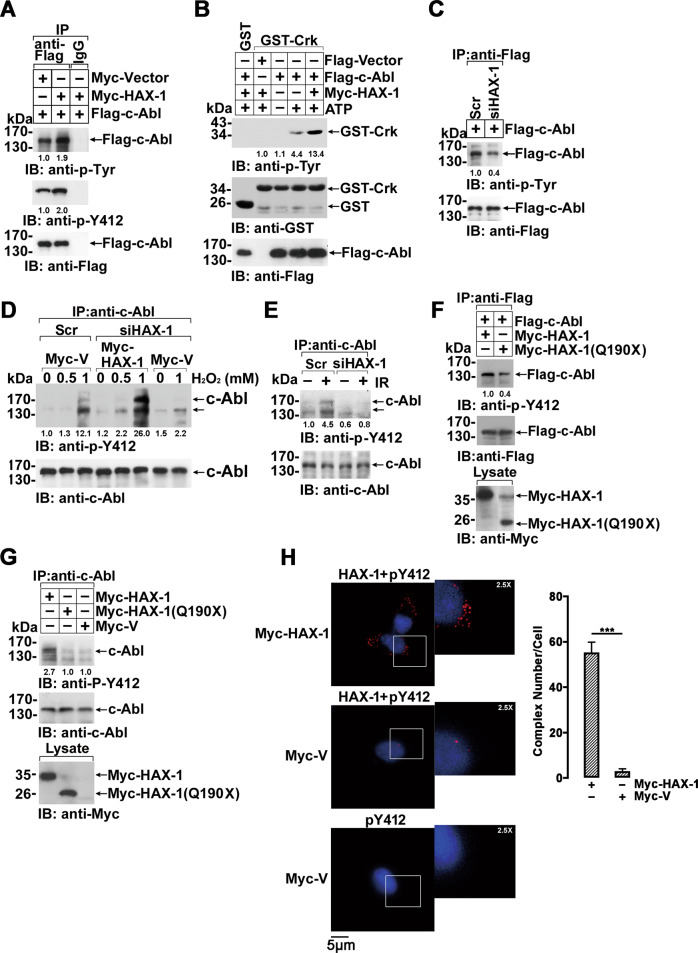
The HAX-1 and c-Abl interaction leads to increased c-Abl tyrosine kinase activity. **A** HEK 293 cells were co-transfected with Flag-c-Abl and Myc-HAX-1 or Myc-vector. Cell lysates were immunoprecipitated with anti-Flag antibody, and the immunoprecipitates were normalized by c-Abl level and immunoblotted with indicated antibodies. **B** In vitro immune complex kinase assay. Flag-c-Abl and Flag vectors were transfected with or without Myc-HAX-1 into HEK 293 cells as indicated. Proteins were purified using anti-Flag antibody-coupled Sepharose beads and then eluted with Flag peptide. In the kinase assay reaction system, the purified recombinant fusion proteins were incubated with GST-Crk fusion protein or GST protein at 30 °C for 30 min. ATP was added to the reaction buffer as indicated. The products were analyzed by SDS-PAGE and Western blot with indicated antibodies. **C** MCF-7 scramble and *HAX-1* knockdown cell lines (MCF-7/siHAX-1) were transfected with Flag-Abl vector. Cell lysates were immunoprecipitated with anti-Flag antibody and analyzed by Western blot with indicated antibodies. The immunoprecipitates were normalized by c-Abl level. **D** The MCF-7 scramble or *HAX-1* knockdown cell line (MCF-7/siHAX-1) was transfected with Myc-HAX-1 or vector control. Before being harvested, cells were treated with the indicated dosage of H_2_O_2_ for 3 h. The cell lysates were analyzed by immunoprecipitation and immunoblotted. The immunoprecipitates were normalized by c-Abl level. **E** The MCF-7 scramble or *HAX-1* knockdown cell line (MCF-7/siHAX-1) were subjected to 10 Gy irradiation. Cell lysates were immunoprecipitated with anti-c-Abl antibody, and immunoblots were probed with indicated antibodies. The immunoprecipitates were normalized by c-Abl level. **F** Flag-c-Abl was co-transfected with Myc-HAX-1 or Myc-HAX-1(Q190X) in 293 cells. Cell lysates were immunoprecipitated with anti-Flag antibody. The immunoprecipitates were normalized by c-Abl level and immunoblotted with indicated antibodies. **G** HEK 293 cells were transfected with Myc-HAX-1, Myc-HAX-1(Q190X) or Myc-vector. Cell lysates were immunoprecipitated with anti-c-Abl antibody. The immunoprecipitates were normalized by c-Abl level and immunoblotted with indicated antibodies. **H** MCF-7 cells transfected with Myc-HAX-1 or Myc-vector plasmids were incubated with target primary antibodies from two different species or with anti-c-Abl antibody as a control. The red spots reveal c-Abl/HAX-1 interaction. Nuclei are stained with Hoechst 33342 (left). Quantification of HAX-1-c-Abl interaction complexes. The number of complexes per cell was counted in at least three fields. Quantifications were given as the mean±S.D. Representative results are shown from experiments repeated three times (right). ***p < 0.001, Student’s *t* test.

Previous studies have demonstrated that c-Abl is activated by several c-Abl binding partners by binding the SH3 and SH2 domains to relieve autoinhibition [37–39]. We then investigated whether c-Abl was activated by HAX-1 association. Normalized by immunoprecipitated c-Abl level, the autophosphorylation of c-Abl was significantly enhanced with the co-expression of HAX-1, including the phosphorylation of Y412, a representative phosphorylation site required for c-Abl kinase activation (Fig. 3A and S3A). The increased catalytic activity of c-Abl kinase was also detected in the presence of HAX-1 by an in vitro kinase assay using GST-Crk (120–225) as a substrate (Fig. 3B and S3B). Accordingly, in MCF-7/*HAX-1* RNAi cells, the phosphorylation of exogenous c-Abl was significantly lower than that in wild-type cells (Fig. 3C and S3C). Moreover, since c-Abl activity is correlated with its Y412 phosphorylation status, the comparison of Y412 should be made based on the equal c-Abl background level. When normalized by the immunoprecipitated c-Abl protein level, c-Abl phosphorylation was hardly detected by *HAX-1* knockdown even with H_2_O_2_ treatment, which indicated that c-Abl kinase could not be effectively activated by H_2_O_2_ without HAX-1 involvement (Fig. 3D, 6th lane, and Fig. S3D). We then rescued HAX-1 expression by concurrent expression of RNAi-resistant *HAX-1* in MCF-7/*HAX-1* RNAi cells. Compared with the control, HAX-1 rescue resulted in enhanced phosphorylation and significant activation of c-Abl kinase in response to H_2_O_2_ stimulation (Fig. 3D, 6th lane, and Fig. S3D). Similarly, an IR-induced increase in Y412 phosphorylation was observed in wild-type MCF-7 cells but not in MCF-7/*HAX-1* RNAi cells (Fig. 3E and S3E). Concordantly, the HAX-1(Q190X) mutant that was not associated with c-Abl failed to activate ectopically expressed or endogenous c-Abl regardless of the presence of stress stimuli (Fig. 3F, G and S3F–S3G). In these experiments, the cellular c-Abl level normalized by beta-Actin in lysates were also detected and shown in the right panel of Figure S3D–S3G. Additionally, in situ PLA showed that the association of HAX-1 with phosphorylated c-Abl was significantly enhanced in HAX-1-overexpressing cells (Fig. 3H). These results collectively demonstrated a new mechanism of c-Abl kinase activation by HAX-1 association, which is also indispensable for oxidative or genotoxic stress-induced c-Abl activation.

### HAX-1 facilitated c-Cbl-mediated ubiquitin-proteasomal degradation of c-Abl kinase

**Fig. 4:**
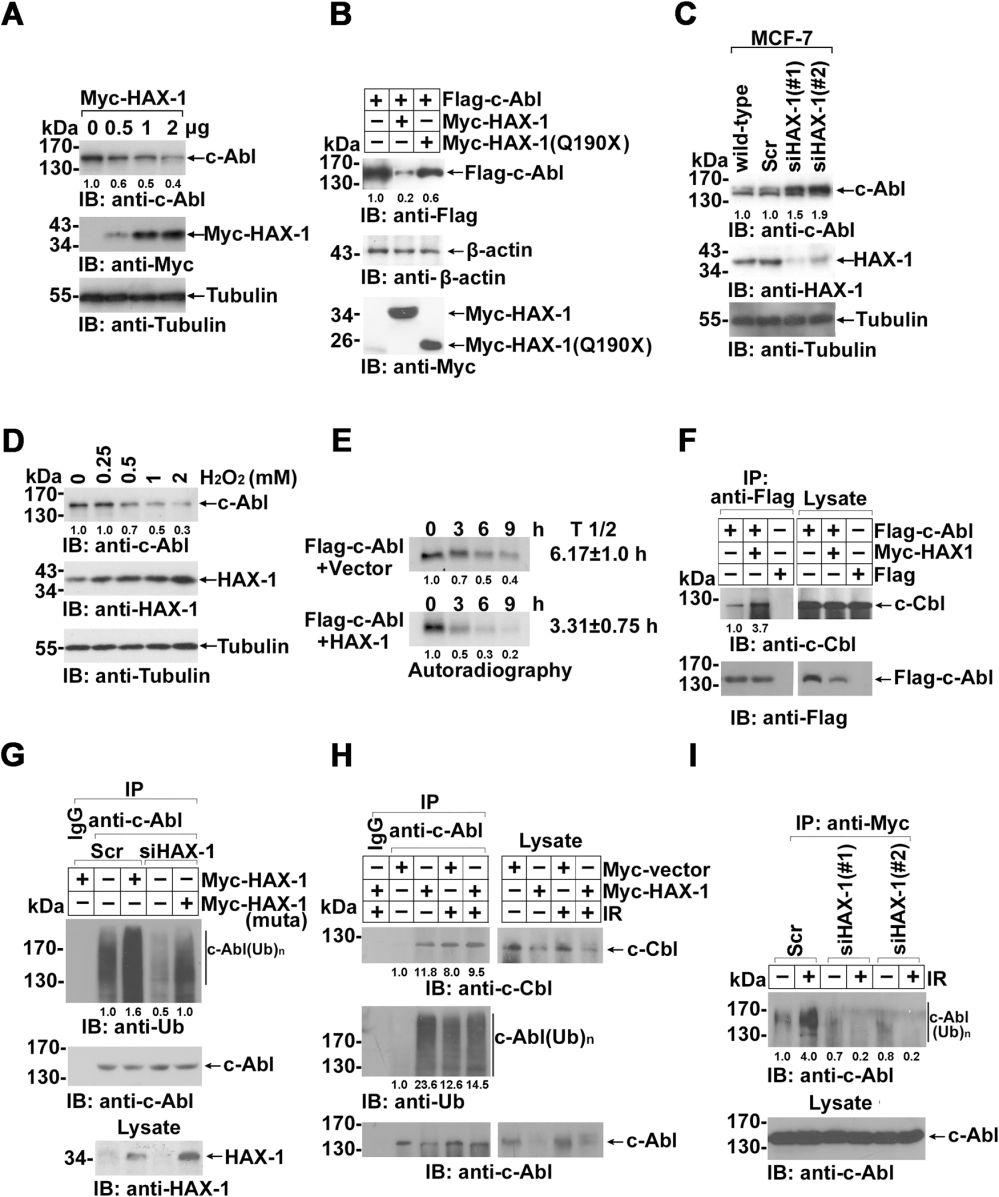
HAX-1 contributes to a reduction in c-Abl expression levels. **A** Western blot analysis with specific antibodies for determination of the levels of total Flag-c-Abl status in HEK 293 cells expressing different dosages of Myc-HAX-1 protein. All cells were transfected with 1 μg Flag-c-Abl, and lanes 2, 3 and 4 were transfected with 0.5, 1.0 and 2.0 μg Myc-HAX-1, respectively. Mock (Lane 1) served as a control in which empty vector was used to transfect the cells. Cellular tubulin was used as an internal control for comparison of protein load in each lane. **B** Western blot analysis of HEK 293 cells transfected with the indicated plasmids. The expression of β-actin served as a loading control. **C** Western blot using anti-HAX-1 antibody identified the MCF-7 clones that were transfected with the *HAX-1*-specific siRNA or scrambled sequence. The expression level of HAX-1 and c-Abl was normalized to the tubulin loading control. **D** HEK 293 cells were treated with increased concentrations of H_2_O_2_ for 3 h and subjected to Western blot analysis. **E** Flag-c-Abl was co-expressed with the Myc-vector or with Myc-HAX-1 in HEK 293 cells. Cells were pulsed with [^35^S] methionine for 45 min, washed and then incubated in [^35^S] methionine-free DMEM for the indicated time. Lysates were immunoprecipitated with anti-Flag antibody and analyzed by SDS-PAGE and autoradiography. The half-life of Flag-c-Abl was calculated according to the intensity of the signals of three independent experiments. **F** Flag-c-Abl was transfected into HEK 293 cells with or without Myc-HAX-1 co-expression. Cell lysates were immunoprecipitated with anti-Flag antibody and immunoblotted with anti-c-Cbl or anti-Flag antibody. **G** MCF-7 scramble cells were transfected with Myc-HAX-1, and *HAX-1* knockdown cells were transfected with a Myc-HAX-1 rescue construct (Muta). For the ubiquitination assay, cells were treated with MG132 (10 μM) 12 h before harvesting. Cell lysates were immunoprecipitated with anti-c-Abl antibody or IgG as a control. The precipitates were analyzed by Western blot using indicated antibodies. **H** MCF-7 cells transfected with or without HAX-1 were IR treated (10 Gy) as indicated. Cell lysates were immunoprecipitated with anti-c-Abl antibody and immunoblotted using indicated antibodies. **I** MCF-7 scramble and two *HAX-1* knockdown clones overexpressing Myc-Ub were IR (10 Gy) treated. After 2 h, cells were harvested, and cell lysates were immunoprecipitated with anti-Myc antibody. The ubiquitination levels of the precipitates were assessed by Western blot.

Activation of c-Abl leads to its notable degradation through the ubiquitin-proteasomal pathway[40]. Accordantly, endogenous or ectopically expressed c-Abl levels were dramatically downregulated by full-length HAX-1 in a dose-dependent manner but not by the truncated mutant HAX-1 (Q190X) (Fig. 4A, B and S4A–S4C) and were upregulated by *HAX-1* knockdown (Fig. 4C and S4D). c-Abl mRNA levels were nearly unchanged by the overexpression or RNAi knockdown of *HAX-1* (Fig. S4E). The stimuli dose-dependent increase in HAX-1 levels induced by H_2_O_2_ evidently contributed to the c-Abl kinase decrease (Fig. 4D and S4F). Furthermore, it was found that the half-life of endogenous c-Abl was ~6.17 h, which was coincident with a previous report[41] but appreciably reduced to ~3.31 h in the presence of ectopically expressed HAX-1, as determined by a [^35^S-Met]-labeled pulse-chase assay (Fig. 4E). Significantly short-lived exogenous c-Abl was also observed with the co-expression of HAX-1 in 293 cells in the presence of the protein biosynthesis inhibitor cycloheximide (CHX) (Fig. S4G). These results indicated that the degradation of c-Abl kinase was notably regulated by HAX-1, accompanied by kinase activation.

In line with the finding that the E3 ubiquitin ligase c-Cbl mediated the degradation of activated c-Abl kinase through the ubiquitin-proteasome pathway [41, 42], the c-Abl kinase abundance in wild-type cells was upregulated to a level similar to that in *HAX-1* RNAi cells after proteasome inhibitor MG132 treatment (Fig. S4H). Furthermore, the binding of c-Abl with its E3 ubiquitin ligase c-Cbl was remarkably strengthened in the presence of HAX-1 (Fig. 4F and S4I). Consequently, the polyubiquitination of c-Abl in *HAX-1* RNAi cells was quite lower than that in wild-type cells and was substantially potentiated by RNAi-resistant *HAX-1* rescue (Fig. 4G and S4J), which was coincident with HAX-1-mediated c-Abl level regulation. Importantly, similar to HAX-1 overexpression, IR stimuli not only activated c-Abl kinase but also greatly enhanced the interaction of c-Abl with c-Cbl and c-Abl polyubiquitination (Fig. 4H and S4K) but failed to induce the ubiquitination of c-Abl in *HAX-1*-deficient cells compared with wild-type or scrambled RNAi cells (Fig. 4I and S4L). These findings collectively indicated that HAX-1 contributes to c-Abl activation and degradation and that the stimulatory factors that activate c-Abl kinase activity are essentially dependent on the presence of HAX-1.

### The anti-apoptosis mediated by HAX-1 was partially dependent on c-Abl activation

**Fig. 5:**
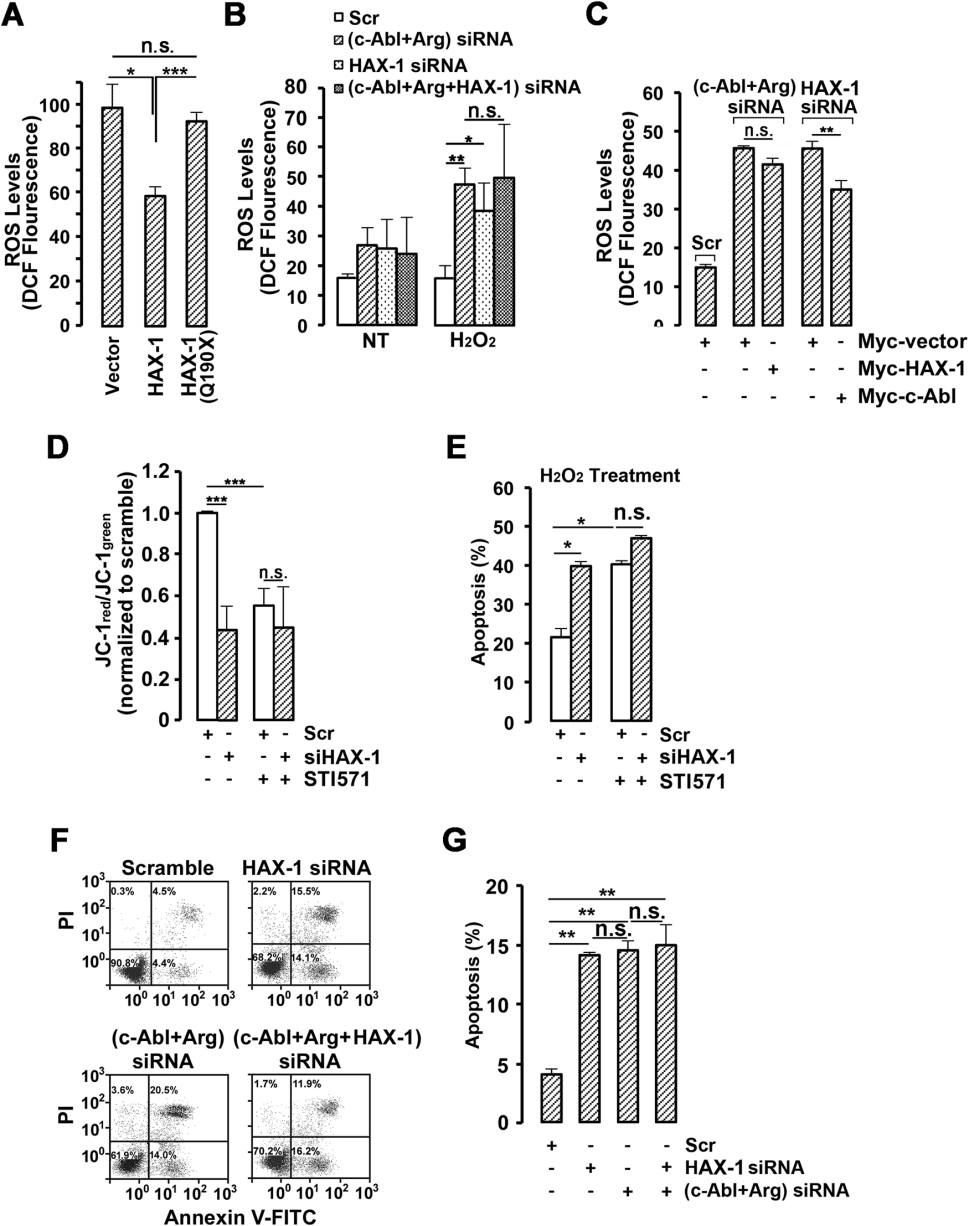
c-Abl is indispensable for the attenuation of cellular ROS levels by HAX-1. **A** SH-SY5Y cells transfected with HAX-1 or HAX-1(Q190X) mutant were treated with 1 mM H_2_O_2_ for 2 h. ROS levels were analyzed by DCFH-DA staining and flow cytometry. The mean frequencies of DCF fluorescence signal intensity were calculated as the mean±S.D. of three independent experiments. n.s., not significant; *p < 0.05, ***p < 0.001, Student’s *t* test. B MCF-7 scramble, *c-Abl/Arg* knockdown, *HAX-1* knockdown or *c-Abl/Arg/HAX-1* triple-gene knockdown cell lines were pretreated with 30 mM 3-AT for 1 h and 1 mM H_2_O_2_ for 2 h as indicated and stained using DCFH-DA. The fluorescence signal intensity of DCF was detected by flow cytometry on a BD Biosciences FACSCalibur. The mean frequencies of DCF fluorescence signal intensity were calculated as the mean±S.D. of three independent experiments. n.s., not significant; *p < 0.01, **p < 0.01, Student’s *t* test. **C** MCF-7 scramble cells, *c-Abl/Arg* knockdown cells overexpressing HAX-1, and *HAX-1* knockdown cells overexpressing c-Abl/Arg were treated with 1 mM H_2_O_2_ for 2 h and stained using DCFH-DA. The fluorescence signal intensity of DCF was detected by flow cytometry on a BD Biosciences FACSCalibur. The mean frequencies of DCF fluorescence signal intensity were calculated as the mean±S.D. of three independent experiments. n.s., not significant; **p < 0.01, Student’s *t* test. **D** MCF-7 scrambled and MCF-7/siHAX-1 cells were treated with 10 μM STI571 for 18 h. Alterations in mitochondrial membrane potential were determined by the ratio of JC-1_red_/JC-1_green_ staining and represented as a ratio of the MCF-7 scramble cells. The mean frequencies of JC-1_red_ and JC-1_green_ were calculated as the mean±S.D. of three independent experiments. n.s., not significant; ***p < 0.001, Student’s *t* test. **E** MCF-7 scrambled and MCF-7/siHAX-1 cells with or without 10 μM STI571 for 18 h were treated with 1 mM H_2_O_2_ for 3 h and analyzed by flow cytometry using FITC-Annexin V (FITC-ANV) and propidium iodide (PI) staining. The percentage of cell death (early and late apoptosis) was identified as ANV^+^PI^+^ on a BD Biosciences FACS Calibur. The mean frequencies of apoptotic cells were calculated as the mean±S.D. of three independent experiments. n.s., not significant; *p < 0.05, Student’s *t* test. **F** MCF-7 scramble, *c-Abl/Arg* knockdown, *HAX-1* knockdown or *c-Abl/Arg/HAX-1* triple-gene knockdown cell lines were analyzed by flow cytometry using FITC-Annexin V (FITC-ANV) and propidium iodide (PI) staining. The percentage of cells undergoing early apoptosis was identified as ANV^+^PI^−^ on a BD Biosciences FACS Calibur. **G** The mean frequencies of apoptotic cells were calculated as the mean±S.D. of three independent experiments. n.s., not significant; **p < 0.01, Student’s *t* test.

We then deeply investigated the biological effects of the HAX-1:c-Abl association. As reported previously, c-Abl kinase activated by ROS stimuli is involved in the elimination of intracellular ROS by regulating the activity of catalase and glutathione peroxidase 1 [28, 43]. Considering that HAX-1 plays important role in neurons apoptosis [9, 44], HAX-1-mediated anti-apoptosis was evaluated not only in MCF-7 cells, but also in neuroblastoma SH-SY5Y cells. HAX-1:c-Abl association and HAX-1-regulated c-Abl expression were observed in SH-SY5Y cells similarly, indicating that ROS scavenging function of HAX-1:c-Abl axis did not limit to a certain cell line (Fig. S5A–S5D). As expected, HAX-1 but not HAX-1 (Q190X) expression dramatically reduced ~40% of cellular ROS in neuron-like SH-SY5Y cells (Fig. 5A). Moreover, *HAX-1* knockdown resulted in increased intracellular ROS levels, similar to the knockdown of *c-Abl/Arg* (Fig. 5B). Notably, compared with the respective knockdown, simultaneous knockdown of both *HAX-1* and *c-Abl/Arg* in the same cell did not lead to a more serious increase in ROS, suggesting that HAX-1 and c-Abl/Arg might regulate cellular ROS levels by the same pathway, in which HAX-1-mediated c-Abl activation functions as an ROS scavenger (Fig. 5B). Accordingly, *HAX-1* knockdown-induced ROS increases could be partly rescued by ectopic expression of c-Abl with constitutive activity (Fig. 5C). In contrast, ectopic expression of HAX-1 in *c-Abl/Arg* knockdown cells had only a minor effect (Fig. 5C).

Furthermore, the mitochondrial membrane potential was dramatically reduced by *HAX-1* knockdown (Fig. 5D). Moreover, treatment with the Abl kinase-specific inhibitor STI571 also resulted in a greatly deleterious effect on mitochondrial membrane integrity in wild-type cells but not in *HAX-1*-deficient cells (Fig. 5D). Accordingly, ~40% of *HAX-1* knockdown cells treated with H_2_O_2_ were subjected to apoptosis, which was much higher than the ~20% apoptosis ratio of wild-type cells with the same treatment. However, the difference in ROS-induced apoptosis between wild-type and *HAX-1* knockdown cells was not observed after STI571 treatment (Fig. 5E), indicating that HAX-1-mediated anti-apoptosis was c-Abl kinase dependent.

Next, we extensively compared H_2_O_2_-induced apoptosis in *HAX*^*−1−*^, *c-Abl/Arg*^*−*/−^, or both-knockdown cells. Similar to STI571 treatment, knockdown also led to substantially increased apoptosis after H_2_O_2_ stimulation compared with wild-type cells (Fig. 5F, G). Notably, knockdown of *c-Abl/Arg* in *HAX-1* RNAi cells did not result in more serious apoptosis, suggesting that the individual rescue of either HAX-1 or c-Abl/Arg expression could not relieve ROS-induced apoptosis in *HAX-1/c-Abl/Arg* triple-knockdown cells (Fig. 5F, G). These findings provide a new mechanism of HAX-1-mediated anti-apoptosis, by which HAX-1 may antagonize ROS-induced cell apoptosis and protect cells from oxidative damage primarily dependent on c-Abl kinase activity.

### HAX-1 insufficiency-induced ROS accumulation and cell death could be rescued by c-Abl activation

**Fig. 6:**
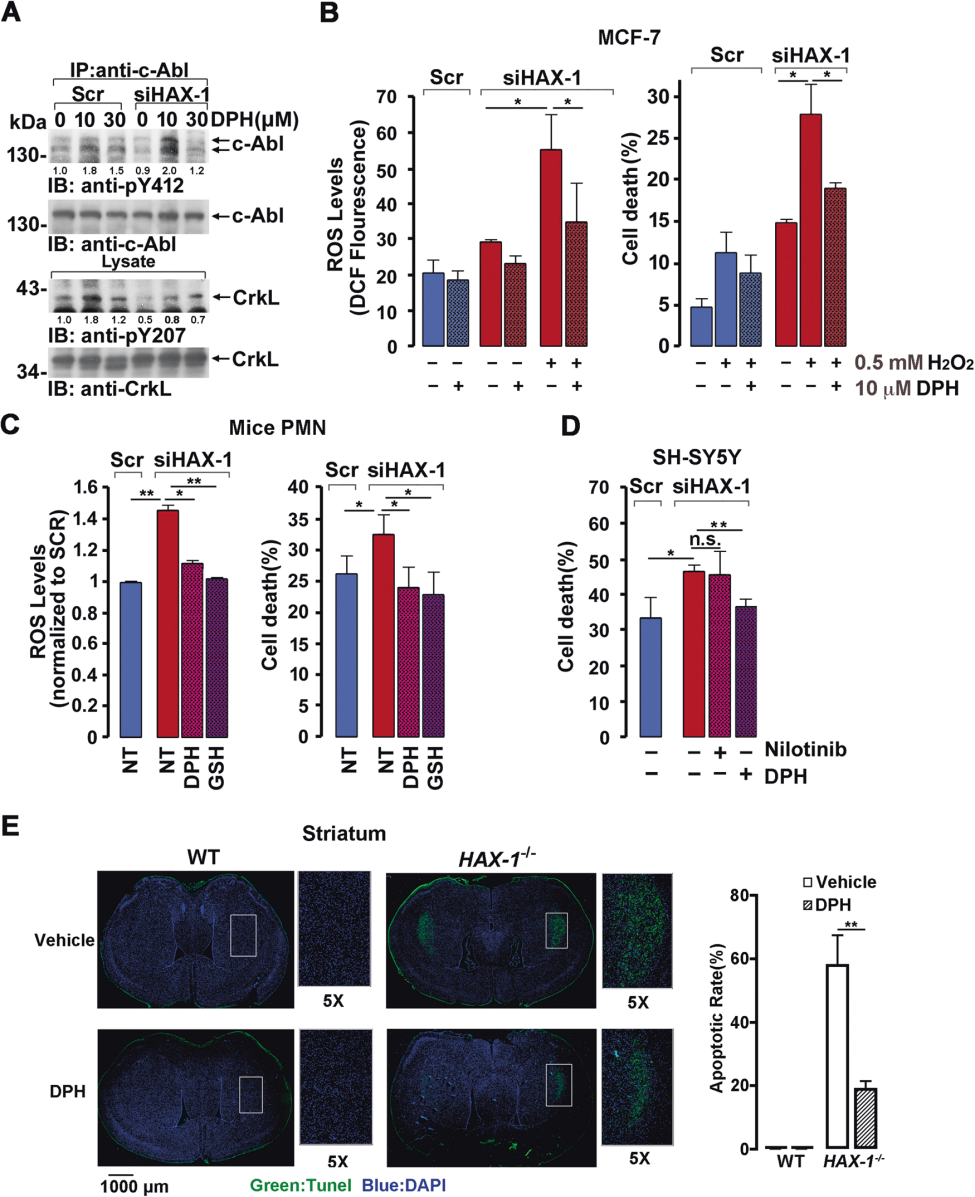
c-Abl activation protected nerve cells from HAX-1 insufficiency-induced ROS accumulation and death. **A** The MCF-7 scramble or *HAX-1* knockdown cell line (MCF-7/siHAX-1) was treated with the indicated concentration of DPH for 12 h, and cell lysates were analyzed by immunoprecipitation and immunoblotted with indicated antibodies. The immunoprecipitates were normalized by c-Abl level. **B** MCF-7 scramble or *HAX-1* knockdown cell lines (MCF-7/siHAX-1) with the indicated H_2_O_2_ (3 h) or DPH (4 h) treatment were subjected to flow cytometry analysis to evaluate ROS levels (left panel) and ratio of cell death (right panel). n.s., not significant; *p < 0.05, Student’s *t* test. **C** PMN-like cells were transfected with lentivirus-HAX-1-siRNA or lentivirus-Scramble-siRNA (MOI = 5), and *HAX-1*-siRNA cells were treated with DPH (10 μM, 12 h) or GSH (1 mM, 3 h). ROS levels and ratio of cell death of all the cells were measured after exposure to 1 mM H_2_O_2_ for 3 h (left panel) or for 8 h (right panel), respectively. *p < 0.05, **p < 0.01, Student’s *t* test. **D** Scramble cells, siRNA-*HAX-1* SY5Y cells, and siRNA-*HAX-1* SY5Y cells treated with nilotinib (5 μM, 18 h) or DPH (10 μM, 18 h) were subjected to 0.5 mM H_2_O_2_ treatment for 2 h, and the death rate was analyzed. n.s., not significant; *p < 0.05, **p < 0.01, Student’s *t* test. **E** WT or *Hax-1* null mice were tail vein injected with DPH or vehicle daily for 30 days, and the striatum were subjected to terminal deoxynucleotidyl transferase-mediated dUTP nick end labeling (TUNEL) staining. TUNEL-positive cells are marked in green, and nuclei are marked with DAPI. Right panel, quantification and statistical analysis of TUNEL-positive cells. **p < 0.01, Student’s *t* test.

We next investigate whether HAX-1 insufficiency-induced ROS accumulation and cell death could be rescued by c-Abl activators. c-Abl showed a compromised activity by *HAX-1* knocking-down, as shown by decreased Y412 autophosphorylation, or Y207 phosphorylation of CrkL, and was activated by DPH (5-(1,3-diaryl-1H-pyrazol-4-yl) hydantoin), a small-molecule reagent that binds to the myristoyl binding site to activate cellular c-Abl, at a concentration of 10 µM (Fig. 6A and S6A-S6B) in scramble as well as *HAX-1* knockdown cells. The cellular c-Abl level normalized by beta-Actin in lysates were also detected and shown in the right panel of Fig. S6A. In accordance, loss of HAX-1 resulted in increased levels of ROS and increased apoptosis in MCF-7 cells, whereas the increase was markedly prevented by treatment with DPH (Fig. 6B). Similarly, mice PMN cells (Fig. 6C) and neuron-like SH-SY5Y cells (Fig. 6D) also exhibited an increased ROS level and increased apoptosis by *HAX-1* knockdown (Fig. 6B, C and S6C). Consistently, treating *HAX-1* RNAi cells with DPH led to a reduction in cellular ROS level, and decreased apoptotic cells, which was not observed by nilotinib treatment, an inhibitor of c-Abl kinase (Fig. 6C, D). This finding indicated that apoptosis caused by HAX-1 insufficiency could be partially rescued by c-Abl activation. Further, in concert with the previous studies showed that *Hax1*-null mice exhibited extensive apoptosis of neurons in the striatum and cerebellum [9], administration of DPH via the tail vein in *Hax-1*-null mice exhibited severely decreased apoptosis to ~20% in the striatum and ~1% in the cerebellum, in comparison to *Hax-1*-null mice injected with vehicle (Fig. 6E and S6D). These results indicate that activation of c-Abl by DPH treatment protected nerve and PMN cells from HAX-1 deficiency-induced apoptosis. And, importantly, glutathione, a ROS scavenger, showed significant protective effect on HAX-1 insufficiency-induced cell apoptosis (Fig. 6C).

## Discussion

HAX-1 was first noted to be a Bcl-2 family member based on its homology with the anti-apoptotic protein [1] and was then found to be a regulator of calcium signaling [45, 46] participating in mitochondria [4] and postmitochondrial apoptosis [47]. HAX-1 was reported to contribute to the processing and activation of the antiapoptotic factor HtrA2 by the mitochondrial protease PARL, thus preventing the accumulation of proapoptotic Bax in the outer mitochondrial membrane [9]. However, the conclusion was later questioned by the observation that HAX-1 lacks BH modules and is peripherally associated with heavy membranes and cannot be mechanistically coupled to PARL because the two proteins are confined in distinct cellular compartments in vivo [48]. These observations suggested that HAX-1 may function in cell apoptosis by as yet unrevealed mechanisms.

c-Abl plays a vital role in the complex regulation of apoptosis, cell proliferation, survival, and cell spreading, including the responses to oxidative stress and DNA damage [18]. It maintained relatively low activity in a normal state and was activated following exposure to many genotoxic agents (e.g., IR, cisplatin, methyl methane sulfonate, mitomycin) and ROS. Protein crystallographic structures of the c-Abl autoinhibited fragment show that the SH3 and SH2 domains are docked onto the surface of the kinase domain distal to the active site and that the kinase is activated through a conformational change in the SH2/SH3 domain [49]. c-Abl interacting proteins may either inhibit (Pag/MSP23 and Aap1) [50, 51] or activate (Crk26 and the DNA-binding protein RFX113) c-Abl [37, 38]. Additionally, oligomerized Abi-1 interacts with c-Abl and contributes to the modulation of autophosphorylation and kinase activity in both normal and oncogenic processes [52]. c-Abl activation also requires and results in autophosphorylation on several tyrosine residues, including Y412 in its activation loop [39]. The crystal structure of the nonphosphorylated Abl kinase domain shows the activation loop tyrosine pointing into the interior of the kinase, making it inaccessible to phosphorylation. The binding of Hck to c-Abl may increase the structural plasticity of the activation loop or induce a conformational change that exposes Y412 for phosphorylation [39], thus resulting in the activation of the kinases. In this paper, HAX-1 was identified as a novel c-Abl binding protein important to a number of the central functions of c-Abl. The interaction was potentiated by c-Abl activating agents such as ion irradiation, CDDP, and especially H2O2 (Fig. 2A, B). Interestingly, similar to the other c-Abl/Arg binding proteins mentioned above, the expression of HAX-1 stimulated autophosphorylation and thus the activation of c-Abl, even though HAX-1 was not found to be a substrate of c-Abl. Importantly, unlike other c-Abl binding proteins, HAX-1 was indispensable for H_2_O_2_-, CDDP- or IR-induced c-Abl autophosphorylation at Y412 (Fig. 3) and activation, suggesting that H_2_O_2_ and the other agents might activate c-Abl by potentiating the interaction of c-Abl and HAX-1 (Fig. 2). To the best of our knowledge, HAX-1 is the first protein demonstrated to activate c-Abl in response to stress stimuli, although the mechanism of how the stimuli regulate HAX-1-c-Abl interactions remains to be unveiled.

Sustained dysregulation or constitutive activation of Abl kinase (such as Bcr-Abl) always resulted in cell death or transformation [53]. To avoid the disaster consequences of Abl hyperactivation, the activity of c-Abl kinase is tightly regulated in the cell. Under normal physiological condition, multiple intramolecular interactions mediate Abl autoinhibition, such as the suppressing configuration formed between SH3, SH2 and kinase domain, as well as the interaction of the myristoylated N-cap with the C-lobe of the kinase domain [18]. Once activated by oxidative or genotoxic stress and other extracellular stimuli, c-Abl is activated rapidly by the association with interacting partners and relieved from the autoinhibited state, resulting in the phosphorylation of downstream substrates and autophosphorylation of c-Abl itself at Y245/Y412. Autophosphorylated c-Abl has much higher kinase activity, and is more prone to be subjected to proteasomal degradation, as a feed-back regulation [41]. As to HAX-1 in this study, it activated c-Abl significantly upon ROS stimulation, thereby promoted c-Abl-mediated activation of catalase and glutathione peroxidase to carry out ROS clearance. As reported previously, a decreased stability of activated c-Abl kinase was also observed not only by stress stimuli but also by HAX-1 overexpression. During this process, the activation of c-Abl and c-Abl-dependent stress-responsive signaling pathway is a more major and rapid event than the subsequent degradation of c-Abl. Although c-Abl activation is transient, its downstream signal transduction lasts until the phosphorylated substrates effector was exhausted. Essentially, it is Abl activation but not degradation that contributes to HAX-involved anti-apoptosis.

Our results also support a central connection between HAX-1 and c-Abl in ROS level modulation. HAX-1-c-Abl interactions were dramatically potentiated by H_2_O_2_, and the expression of HAX-1 strikingly enhanced H_2_O_2_-induced c-Abl kinase activation (Figs. 2, 3). Similar to *c-Abl/Arg* knockdown, downregulation of HAX-1 expression conferred significant increases in intracellular ROS levels, which were partially rescued by c-Abl overexpression. Simultaneous knockdown of *HAX-1* and *c-Abl/Arg* kinases failed to show any synergizing effect, supporting HAX-1 and c-Abl/Arg regulation of cellular ROS by the same pathway (Fig. 5). Similarly, HAX-1 controlled cytoplasmic oxidative stress-induced apoptosis by activating c-Abl and Arg, which were shown to activate antioxidative enzymes, with the subsequent elimination of H_2_O_2_ or other reactive oxidative species (ROS), generating a protective effect against oxidative stress [28, 43]. It has been reported that HAX-1 blocks cell apoptosis by inhibiting the activation of the initiator caspase-9 and death caspase-3 [10, 54]. According to our study, these contributions may be mediated through the regulation of cellular ROS levels by HAX-1 because increasing ROS levels could also activate caspase-9/3 [55, 56].

Loss-of-function mutations of HAX-1 resulted in Kostmann disease, an inherited severe congenital neutropenia syndrome (SCN)[14]. HAX1-deficient neutrophils in SCN patients, but not SCN neutrophils expressing functional HAX-1, showed evidence of enhanced production of ROS. Neutrophils isolated from the patient exhibited spontaneous apoptosis and loss of inner mitochondrial membrane potential, which were further enhanced upon treatment with hydrogen peroxide [57, 58]. HAX-1 was also shown to be protective in MCF-7 cells and neuron-like SHY5Y cells from H_2_O_2_-induced cell death. Recently, it was observed that HAX-1 negatively regulates integrin-mediated adhesion that affects uropod detachment and neutrophil chemotaxis, a process that may be key to the pathogenesis of congenital neutropenia syndromes, such as Kostmann disease [15]. Similarly, *c-Abl/Arg* knockdown resulted in elevated ROS levels. c-Abl also regulates human neutrophil chemotactic activity [59, 60], and the Abl kinase selective inhibitor STI571 can induce neutropenia [61]. Since HAX-1 activates c-Abl to downmodulate cellular ROS levels, SCN neutrophils, where HAX-1 is often functionally mutated or not expressed [14, 62], will be compromised for c-Abl activation, resulting in cellular ROS accumulation and neutrophil death. HAX-1-modulated c-Abl activation may be responsible for the regulation of myeloid cell migration and has a likely role in the pathogenesis of SCN. Our findings underscore the important coordinated role of HAX-1 and c-Abl and form a foundation for further study of critical pathways in cellular stress responses, and patients suffered from Kostman disease may be benefited from c-Abl activators such as DPH, or ROS scavengers.

## Materials and methods

### Plasmids and Generation of *HAX-1* Mutants

Flag-tagged vectors for expressing c-Abl, c-Abl (K290R), and HAX-1 were constructed by cloning the full-length human *HAX-1* (NM_006118) genes into the pcDNA3 vector (Invitrogen,V795-20). Myc-tagged expression plasmids were prepared by cloning into the pCMV-Myc vector (Clontech, 635689). GST fusion proteins were generated by expression in pGEX4T-2-based vectors (Amersham Biosciences Biotech Inc.) in *Escherichia coli* BL21 (DE3).

Deletion mutants were generated by PCR amplification using *HAX-1* plasmid as a template, and the following sets of primers: (i) sense primer-1: 5′ -CGGGATCCATGCTTAAGTATCCAGATAGTCACCAG-3′ and antisense primer-1: 5′-GAAGATCTCTACCGGGACCGGAACCAACGT-3′ were used for generation of a HAX-1 lacking the N-terminal 128 amino acids which contains BH1, BH2 as well as PEST motifs, (ii) sense primer-2: 5′ -GCGAATTCGCATGAGCCTCTTTGATCTCTTCCGGG-3′ and antisense primer-2: 5′-GAAGATCTTTATGAGTCCCGAAGTGTCTGTC-3′ were used for amplification of a HAX-1 lacking the C-terminal 151 amino acids. All sense primers carried an EcoRI recognition site, and all antisense primers contained a BglII site for insertion into the pCMV-myc vector and generation of Myc-tagged fusion peptides. The primer 5′-TGATCTTGATTCCTAGGTTTCCCAGGAGGG-3′ was used to construct the HAX-1 Q190X mutant. The authenticity of all constructs was verified by DNA sequencing.

### Cells and transfection

Human embryonic kidney (HEK) 293 cells, the human breast adenocarcinoma cell line MCF-7 and the human neuroblastoma cell line SH-SY5Y were obtained from ATCC (https://www.atcc.org/). The cells were verified and were free of mycoplasma contamination based on the results of the Mycoplasma Stain Assay Kit (Beyotime). Cells were grown in Dulbecco’s modified Eagle’s medium (DMEM, Sigma,RNBJ5741) supplemented with 10% heat-inactivated fetal bovine serum (Tecono, F801-500), 2 mM L-glutamine, 100 units/ml penicillin, and 100 μg/ml streptomycin. Transient transfections were performed with Lipofectamine 2000 (Invitrogen, 11668019). Cells were treated with STI571 (imatinib, Novartis, 220127-57-1), cisplatin (Sigma, 15663-27-1), H_2_O_2_(Sigma, 7722-84-1), MG132 (Sigma, M8699) or lactacystin (Biomol, HY-16594) as indicated.

### Immunoprecipitation and immunoblot analysis

Cell lysates were prepared in lysis buffer (50 mM Tris-HCl, pH 7.5, 150 mM NaCl, 1% (v/v) Nonidet P-40, 0.5 mM EDTA) supplemented with complete protease inhibitor cocktail (Roche,04693132001). Soluble protein was subjected to immunoprecipitation with anti-Flag antibody (agarose-conjugated, M-2, Sigma-Aldrich, A2220), anti-c-Abl antibody (K-12, Santa Cruz Biotechology, Cat# SC-131), or anti-rabbit IgG antibody (agarose-conjugated, Sigma-Aldrich). An aliquot of the total lysate (5%, v/v) was included as a control. Immunoblot analysis was performed with anti-HAX-1 antibody (FL-279, Santa Cruz Biotechology, sc-28268), anti-c-Abl antibody (K12 rabbit polyclonal antibody, Santa Cruz), anti-c-Cbl antibody (Santa Cruz, sc-1651), anti-tubulin antibody (Sigma-Aldrich, Cat# T9822), anti-β-actin(Santa Cruz,Cat# SC-1616), or HRP conjugated anti-Flag antibody (Sigma-Aldrich, Cat# A8592), anti-Myc antibody (Santa Cruz, Cat# SC-40), anti-Ub antibody (Biomol, NSJ-R30490) and anti-phosphotyrosine antibody (4G10, Millipore, Cat# 16-105). The antigen-antibody complexes were visualized by chemiluminescence (ECL, Millipore, WBKLS0500). Data shown are representative of three independent experiments. The relative intensity of WB bands was quantified by gray scanning and represented as mean±S.D. of three independent analysis. n.s., not significant; *p < 0.05, **p < 0.01, ***p < 0.001, Student’s *t* test.

### Glutathione transferase (GST)-pull down assay

GST fusion proteins were generated by expression in pGEX4T-2 (Amersham Pharmacia Biotech Inc.) vectors in *E. coli* BL21 (DE3) (Novagen) and purified by affinity chromatography using glutathione Sepharose beads (GE Healthcare). Cell lysates were incubated with 2 μg of GST, GST-HAX-1 and GST-c-Abl-SH3 immobilized on the beads for 2 h at 4°C. The adsorbates were washed with lysis buffer and then subjected to SDS-PAGE and immunoblot analysis. An aliquot of the total lysate (2%, v/v) was included as a loading control. Data shown are representative of three independent experiments.

### Pulse-chase Assays

HEK 293 cells transfected with Flag-c-Abl/Myc-Vector or Flag-c-Abl/Myc-HAX-1 were washed with Met/Cys-free DMEM (Gibco), and then incubated with Met/Cys-free DMEM containing 10 μCi/ml [^35^S] methionine (Amersham Biosciences Biotech Inc.) for 45 min. The cells were then washed and cultured in complete DMEM containing 10% heat-inactivated FBS and harvested at the indicated time points. Anti-Flag immunoprecipitates were subjected to SDS-PAGE and autoradiography. The bands were then excised and subjected to liquid scintillation counting for quantification. Data shown are representative of three independent experiments.

### c-Abl tyrosine kinase assay

Purified recombinant GST-HAX-1 (0.5 μg) or GST-Crk was incubated with recombinant c-Abl (0.02 μg; Upstate Biotechnology Inc.) in kinase reaction buffer (20 mM HEPES (pH 7.5), 75 mM KCl, 10 mM MgCl_2,_ and 10 mM MnCl) containing 2 mM ATP for 30 min at 37 °C. The reaction products were analyzed by SDS-PAGE and immunoblot. A fusion protein GST-Crk containing the c-Abl phosphorylation site in the adapter protein CRK was used as a specific substrate to assay c-Abl kinase. Data shown are representative of three independent experiments. The relative intensity of WB bands was quantified by gray scanning and represented as mean±S.D. of three independent analysis. ***p < 0.001, Student’s *t* test.

### Silencing *HAX-1* with short interfering RNAs (siRNA)

The *HAX-1* siRNA sequences were selected by using an siRNA selection program. Synthesized and purified oligonucleotides were annealed and cloned into the pSUPER-retro-neo plasmid (Oligo Engine, Inc.). The *HAX-1* siRNA construct was named pSuper-GFP-HAX-1-siRNA. The scrambled control plasmid (pSuper-GFP-C) encodes an shRNA that did not match any sequence found in the human genome database. The sequence used for constructing pSuper-GFP-HAX-1 siRNA was 5′-GATCCCCAACCAGAGAGGACAATGATCTTTCAAGAGAAGATCATTGTCCTCTCTGGTTTTTTTA-3′. The scrambled sequence used as a control was 5′-GATCCCCAGAGCGAGAGCCTCTATATTTCAAGAGAATATAGAGGCTCTCGCTCTTTTTTA-3′. The primer 5′-CCTAGAACCAGGGAGGATAATGACCTTGATTCCC-3′ was used to construct a *HAX-1* mutant that could not be influenced by RNAi. Stable cell lines were obtained by treating the cells with 800 μg/mL G418 and identified by GFP and HAX-1 protein expression.

### Apoptosis analysis by FITC-Annexin V and propidiumiodide staining

To monitor the association of HAX-1 with cell apoptosis, MCF-7 cells were analyzed by flow cytometry using FITC-Annexin V (FITC-ANV) and propidium iodide (PI) staining. Apoptotic cells were identified as ANV^+^PI^−^ on a BD Biosciences FACSCalibur. The mean frequencies of apoptotic cells were calculated as the mean±S.D. of three independent experiments. n.s., not significant; *p < 0.05, **p < 0.01, Student’s *t* test.

### Detection of ROS

Cells were harvested using trypsin, washed twice with serum-free DMEM, and then incubated with DCFH-DA (Beyotime) at a final concentration of 10 μM for 20 min at 37 °C in the dark. Then, the cells were washed once with serum-free DMEM and analyzed immediately by flow cytometry. Data were shown as mean±S.D. of three independent analysis. n.s., not significant; *p < 0.05, **p < 0.01, ***p < 0.001, Student’s *t* test.

### Measurement of mitochondrial membrane potential by JC-1

Cells were harvested using trypsin and stained with JC-1 (Beyotime) according to the manufacturer’s protocol. The ratio of JC-1 _aggregate_/JC-1 _monomer_ was determined by calculating the mean FL2/FL1 fluorescence detected by flow cytometry. Data were shown as mean±S.D. of three independent analysis. n.s., not significant; ***p < 0.001, Student’s *t* test.

### In situ proximity ligation assay (in situ PLA)

Duolink in situ PLA (Duolink Detection kit, Olink Bioscience, Uppsala, Sweden) was used to detect interactions between HAX-1 and c-Abl. Briefly, MCF-7 cells plated on glass coverslips were treated with cisplatin for 24 h and fixed as described above. The fixed cells were incubated with rabbit anti-HAX-1 and mouse anti-c-Abl (Sigma-Aldrich) primary antibodies. The Duolink system provides oligonucleotide-labeled secondary antibodies (PLA probes) to each of the primary antibodies that, in combination with a DNA amplification-based reporter system, generate a signal only when the two primary antibodies are in close enough proximity. The signal from each detected pair of primary antibodies was visualized as a spot (see the manufacturer’s instructions). Slides were evaluated using an LSM 510 META confocal microscope (Carl Zeiss). Cell images obtained were exported using the Zeiss LSM Image Browser (Carl Zeiss) in TIF format for further analysis. Interactions per cell were determined with the Duolink image tool, which was developed by Olink Bioscience and was counted in at least three fields. Quantifications were given as the mean±S.D. n.s., not significant; **p < 0.01, ***p < 0.001, Student’s *t* test. Representative results are shown from experiments repeated three times.

### Confocal microscopy

Cells were fixed with 4% paraformaldehyde for 20 min, permeabilized with 0.2% Triton X-100 for 10 min at room temperature, and nonspecifically blocked with PBS buffer containing 1% goat serum for 1 h. The cells were then incubated with primary antibody for 1 h and secondary antibody for another 1 h at room temperature. Nuclei were attained with Hochest33342. Images were randomly obtained using the Hochest33342 channel to avoid bias in the selection of cells with particular phenotypes before other channels were used for imaging.

### Mice

C57 *Hax-1*^*−/+*^ mice were purchased from Cyagen Biosciences Inc., and heterozygous mice were crossed to generate homozygosity. All mice were bred and maintained in the animal facility of the Military Medical Research Institute according to the institutional and national guidelines for animal care and use. The animal studies were approved by the Institutional Ethics Committee of Military Medical Science.

### Apoptosis detection of neurons in the striatum and cerebellum

Twenty-eight-day-old WT or *Hax-1* null female mice (eight mice per group) were randomly divided into groups before the experiment, then tail vein injected with DPH (5 μg) or vehicle daily for 30 days. The mice were sacrificed and immediately perfused with 4% paraformaldehyde. The striatum and cerebellum were extracted and fixed in 4% paraformaldehyde and then subjected to terminal deoxynucleotidyl transferase-mediated dUTP nick end labeling (TUNEL) staining according to the standard protocol of the Roche In Situ Cell Death Detection Kit. TUNEL-positive cells are marked in green, and nuclei are marked with DAPI. Representative images are shown. The TUNEL assays were performed by Wuhan Servicebio Technology Co., Ltd. Investigators were blinded to the order of samples.

### Statistical analysis

All experiments were replicated at least three times, and Statistical analysis was carried out using unpaired wo-tailed Student’s *t* test by GraphPad Prism 7. No data were excluded from the analyses unless indicated. Data were considered significant when p < 0.05.

## Supplementary information

Supplementary Information file

Supplementary Figure 1

Supplementary Figure 2

Supplementary Figure 3A-E

Supplementary Figure 3F-G

Supplementary Figure 4A-F

Supplementary Figure 4G-L

Supplementary Figure 5

Supplementary Figure 6

Supplementary Figure Legends

checklist

## Data Availability

All data generated or analyzed during this study are included in the article and its supplementary files, and available from the corresponding author on reasonable request.
