# Supplementary material for: Anti-apoptotic HAX-1 suppresses cell apoptosis by promoting c-Abl kinase-involved ROS clearance
Source: Cell Death Dis. 2022 Apr 4;13(4):298. doi: 10.1038/s41419-022-04748-2 (PMC8979985; doi:10.1038/s41419-022-04748-2)
Supplement: Supplementary file 1 — Supplementary Information file [file 41419_2022_4748_MOESM1_ESM.pdf]

Supplementary Figure 1

S1A

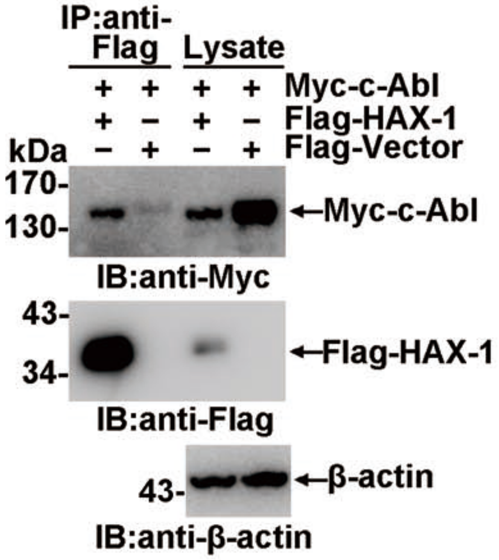

S1B

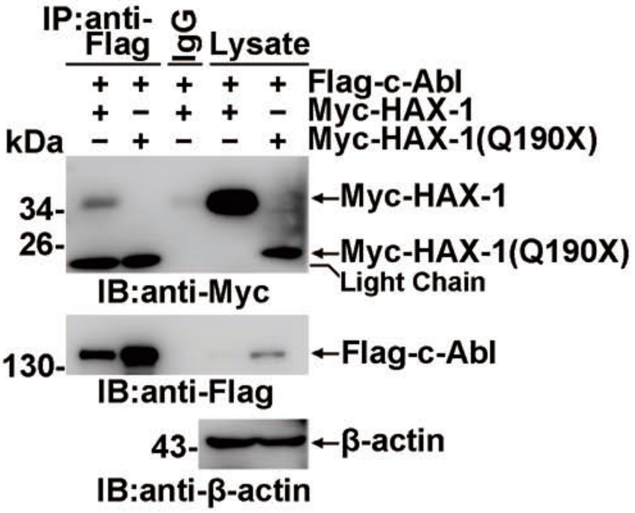

Supplementary Figure S1. Association of HAX-1 with c-Abl. (S1A)-(S1B) HEK 293 cells co-transfected with indicated plasmids were analyzed by immunoprecipitation and immunoblotting with indicated antibodies. Whole lysates (2% v/v) normalized by β-actin level were used as controls to confirm HAX-1 and c-Abl expression.

Supplementary Figure 2

S2A

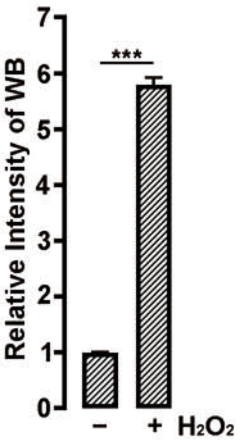

Supplementary Figure S2. Interaction of c-Abl with HAX-1 upon ROS stimulation. (S2A) The relative intensity of WB bands in Fig. 2F was quantified by gray scanning and represented as mean±S.D. of three independent analysis. ***p<0.001, Student's t test.

Supplementary Figure 3

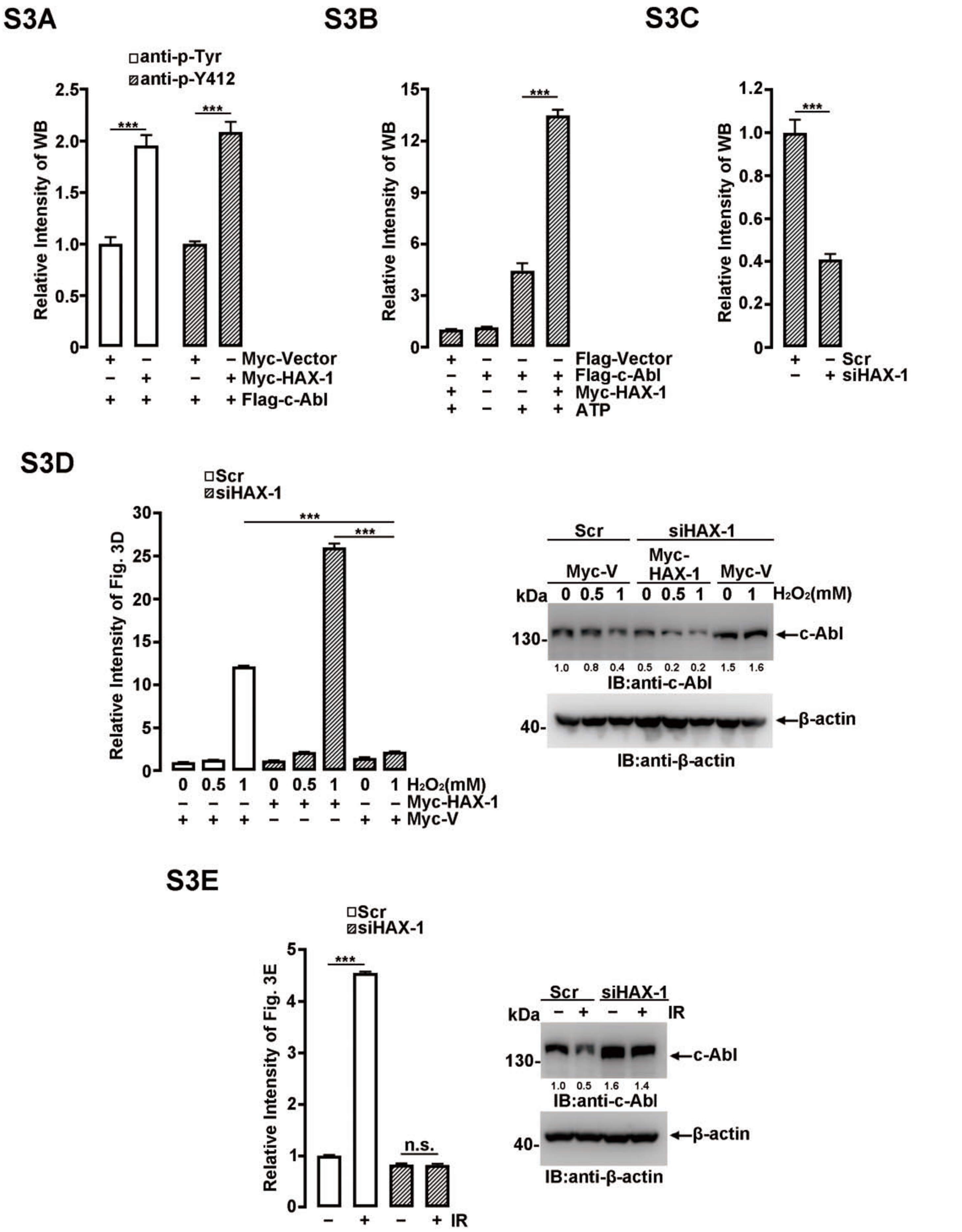

Supplementary Figure 3

S3F

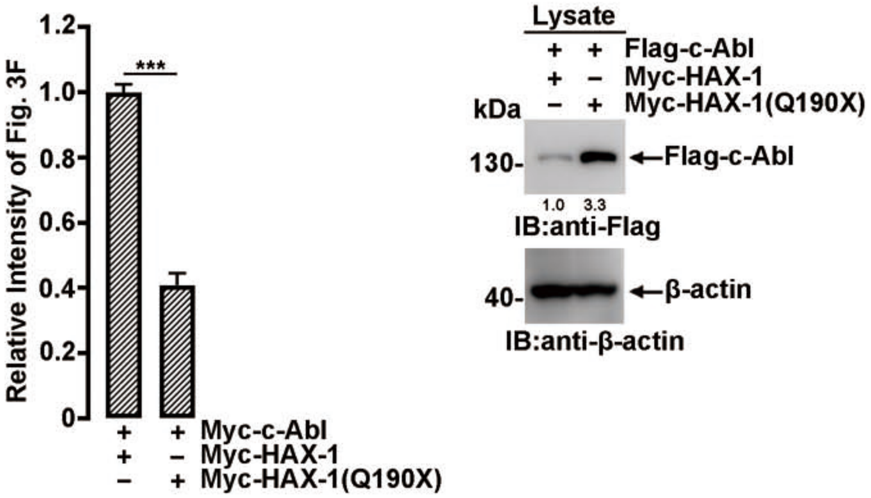

S3G

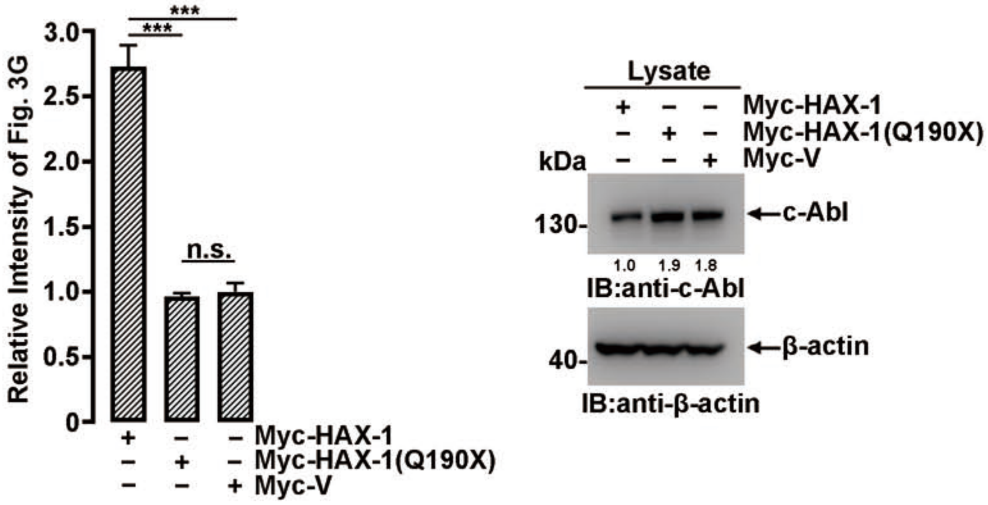

Supplementary Figure S3. The HAX-1 and c-Abl interaction leads to increased c-Abl tyrosine kinase activity. (S3A)-(S3G) The relative intensity of WB bands in Fig. 3A-3G was quantified by gray scanning and represented as mean±S.D. of three independent analysis. n.s., not significant; ***p<0.001, Student's t test. The right panel of S3D-S3G showed the Western blot analysis of cellular c-Abl levels normalized by beta-Actin in lysates of Fig. 3D-3G.

S4A

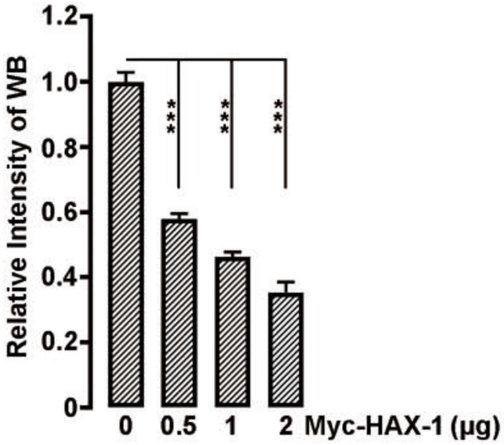

S4B

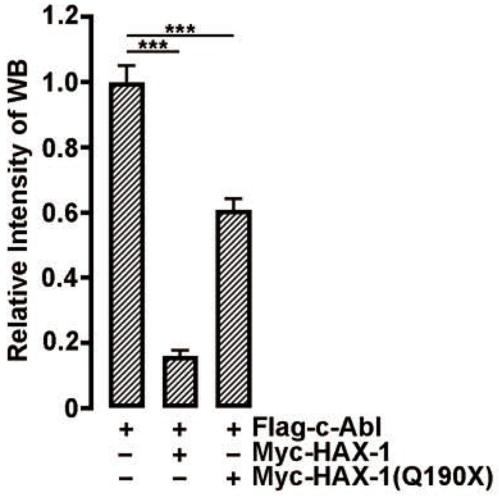

S4C

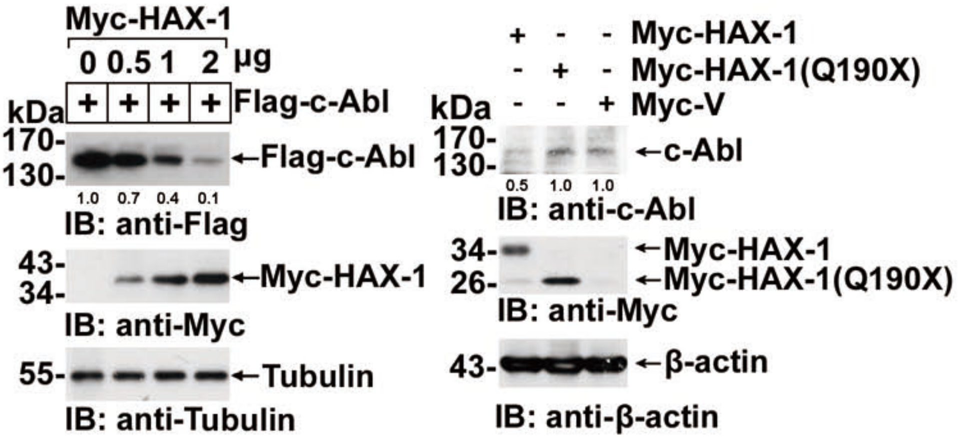

S4D

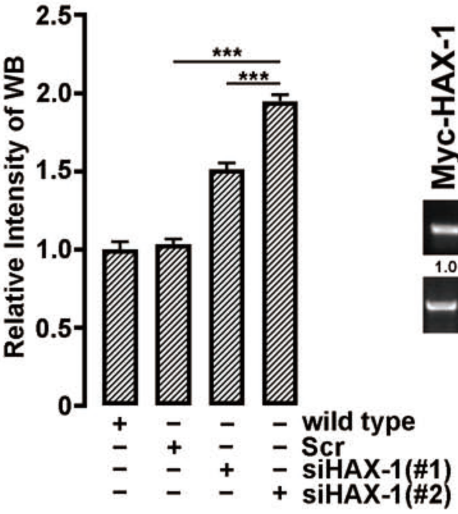

S4E

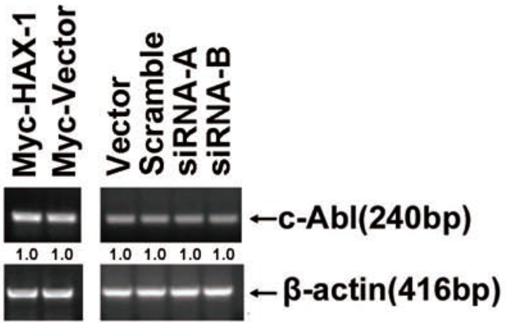

S4F

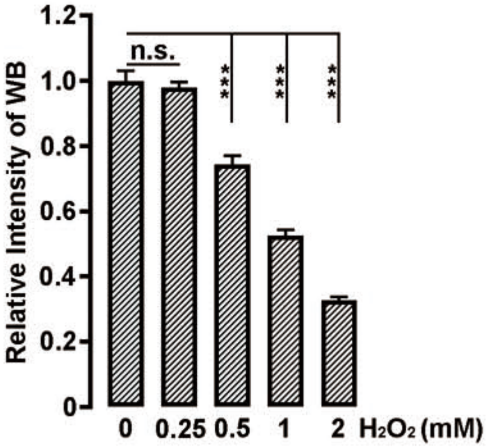

Supplementary Figure S4. HAX-1 contributes to a reduction in c-Abl expression levels. (S4A)-(S4B) The relative intensity of WB bands in Fig. 4A-4B was quantified by gray scanning and represented as mean±S.D. of three independent analysis. ***p<0.001, Student's t test. (S4C) Endogenous c-Abl protein was detected by Western blot analysis in HEK 293 cells transfected with the indicated dosage of Myc-HAX-1. The expression of tubulin served as a loading control (left). HEK 293 cells transiently transfected with Myc-HAX-1, Myc-HAX-1 (Q190X) or Myc-vector plasmids were subjected to Western blot analysis using indicated antibodies (right). (S4D) The relative intensity of WB bands in Fig. 4C was quantified by gray scanning and represented as mean±S.D. of three independent analysis. ***p<0.001, Student's t test. (S4E) MCF-7 cells transfected with Myc-HAX-1 or Myc-vector, MCF-7 scramble and two MCF-7/*HAX-1* siRNA cell lines were subjected to polymerase chain reaction (PCR) to evaluate c-Abl mRNA levels, with β-actin mRNA as a loading control. (S4F) The relative intensity of WB bands in Fig. 4D was quantified by gray scanning and represented as mean±S.D. of three independent analysis. n.s., not significant; ***p<0.001, Student's t test.

Supplementary Figure4

S4G

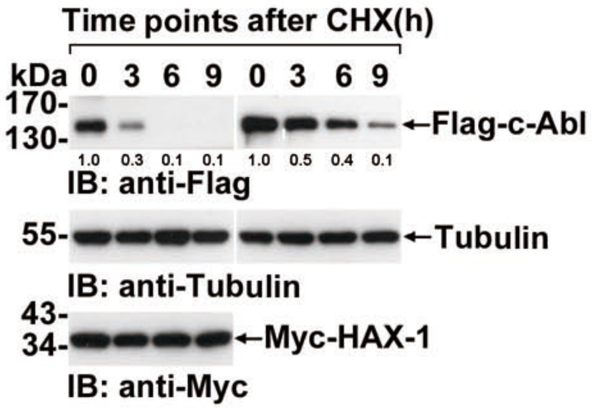

S4H

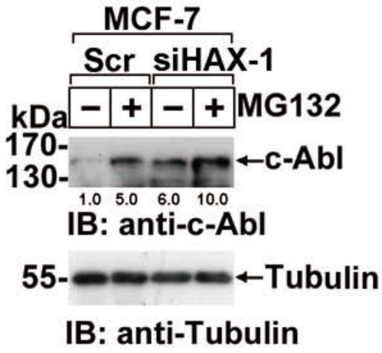

S4I

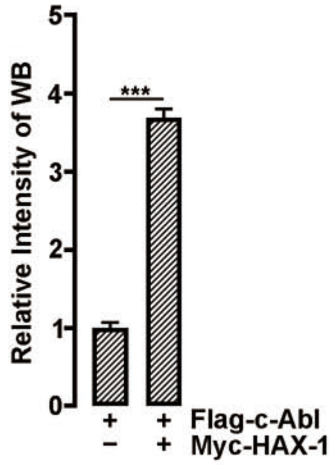

S4J

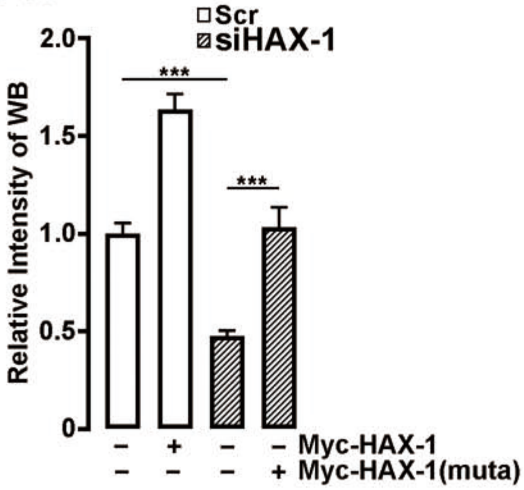

S4K

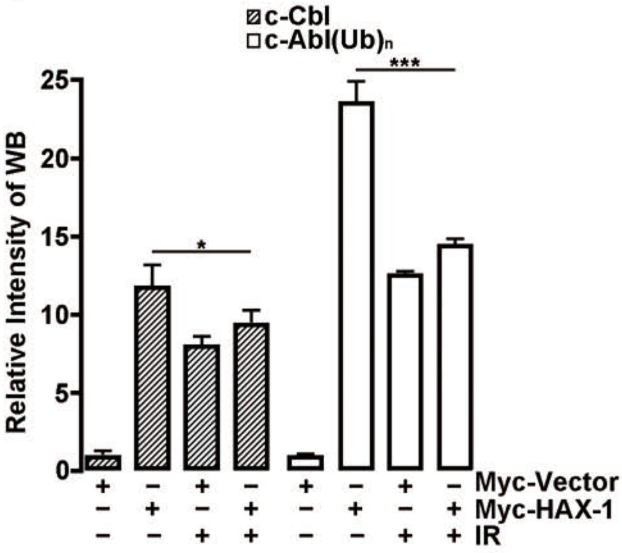

S4L

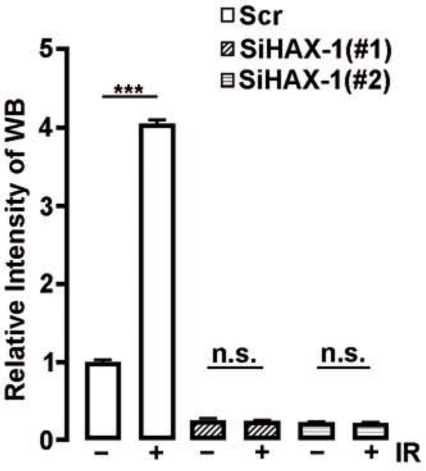

(S4G) HEK 293 cells transiently transfected with Flag-c-Abl and Myc-HAX-1 or empty vector were treated with the protein synthesis inhibitor cycloheximide (CHX, 100 μg/ml) for the indicated time and then subjected to Western blot analysis using indicated antibodies. (S4H) MCF-7 scramble and MCF-7/*HAX-1* siRNA cells were treated with or without the proteasome inhibitor MG132 (10 μM) for 12 h. Cell lysates were subjected immunoblotting with indicated antibodies. (S4I)-(S4L) The relative intensity of WB bands in Fig. 4F-4I was quantified by gray scanning and represented as mean±S.D. of three independent analysis. n.s., not significant; *p<0.05, ***p<0.001, Student's t test.

Supplementary Figure 5

S5A

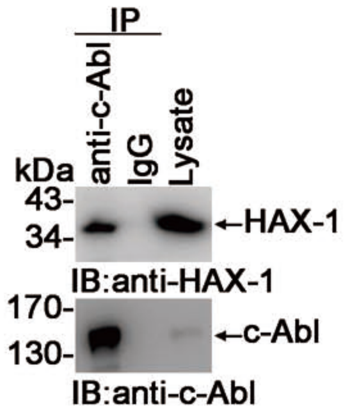

S5B

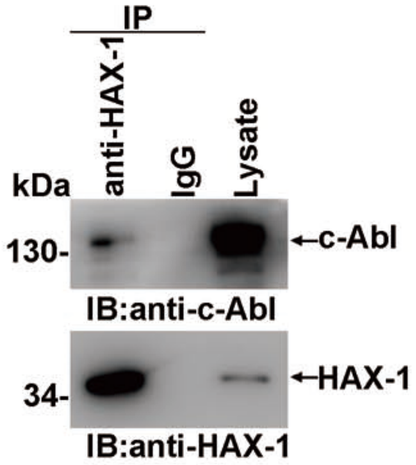

S5C

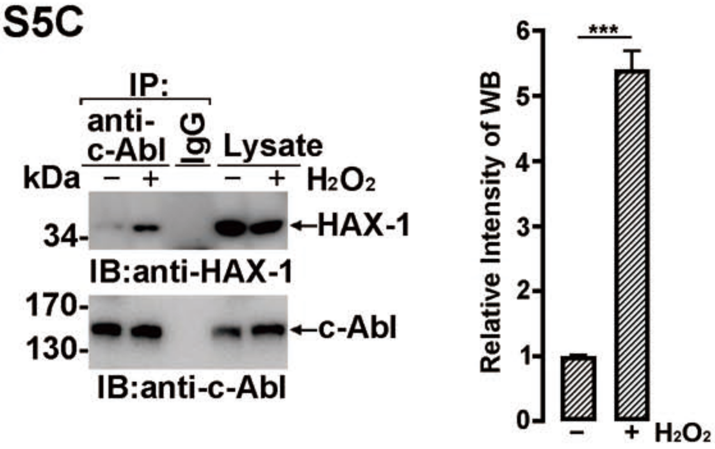

S5D

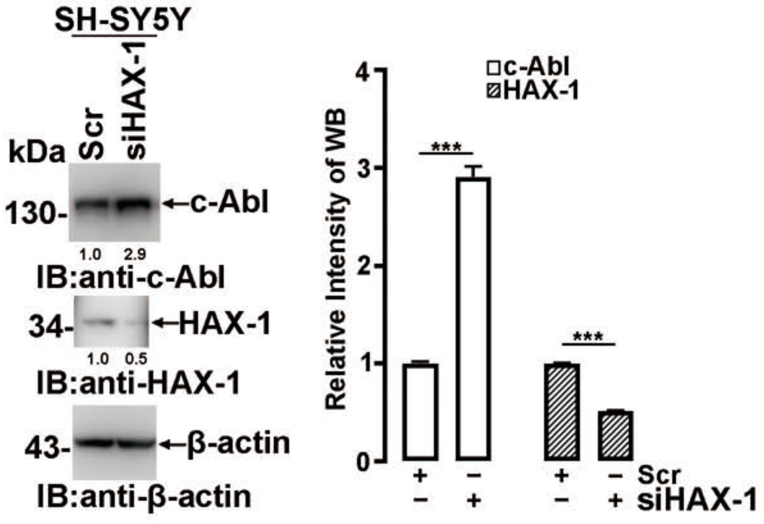

Supplementary Figure S5. The existence of HAX-1:c-Abl axis in neuroblastoma SH-SY5Y cell. (S5A)-(S5B) Total lysates from SH-SY5Y cells were subjected to anti-c-Abl (S5A) or anti-HAX-1 (S5B) immunoprecipitation, and analyzed by immunoblotting. (S5C) Lysates from SH-SY5Y cells treated with or without H2O2 (1 mM, 3 h) were subjected to anti-c-Abl immunoprecipitation, and the immunoprecipitates were analyzed by immunoblotting (left). The relative intensity of WB bands was quantified by gray scanning and represented as mean±S.D. of three independent analysis. ***$p<0.001$, Student's t test (right). (S5D) SH-SY5Y cells were transfected with the HAX-1 siRNA or scramble siRNA (as a control), and were analyzed by immunoblotting (left). The relative intensity of WB bands was quantified by gray scanning and represented as mean±S.D. of three independent analysis. ***$p<0.001$, Student's t test (right).

Supplementary Figure 6

S6A

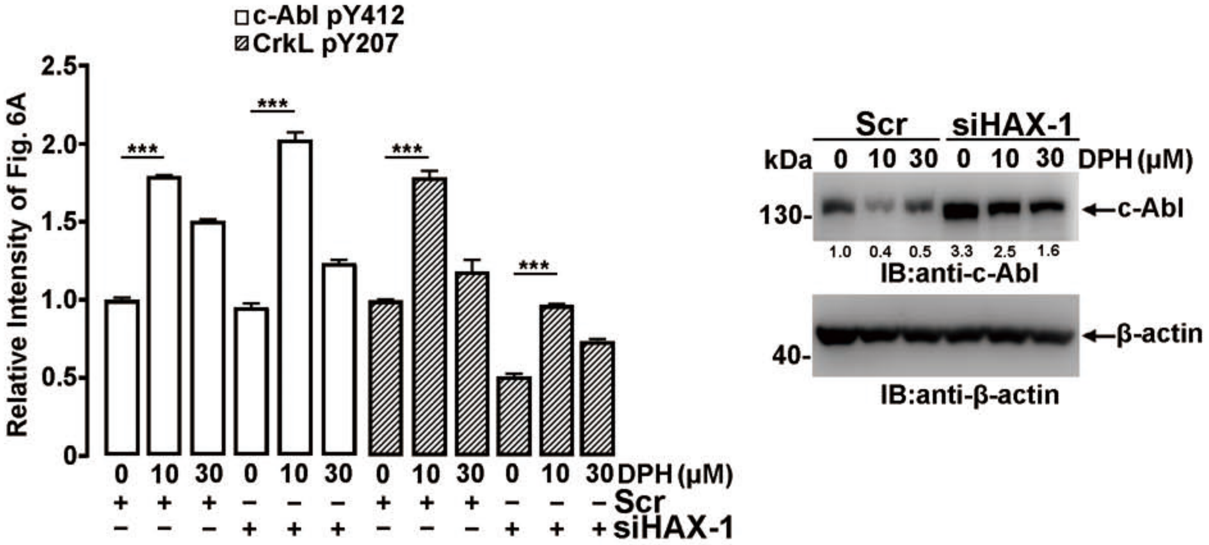

S6B

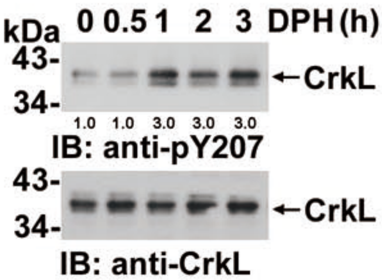

S6C

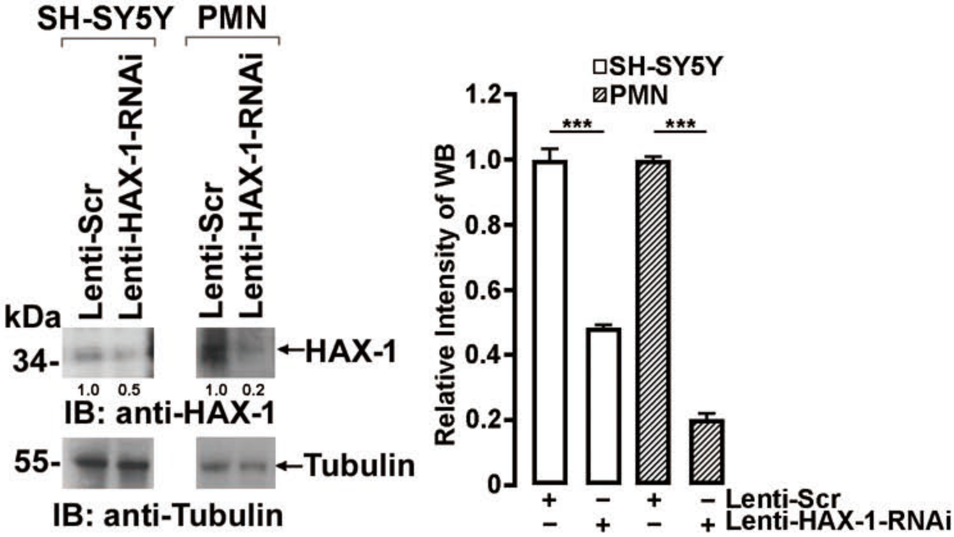

S6D

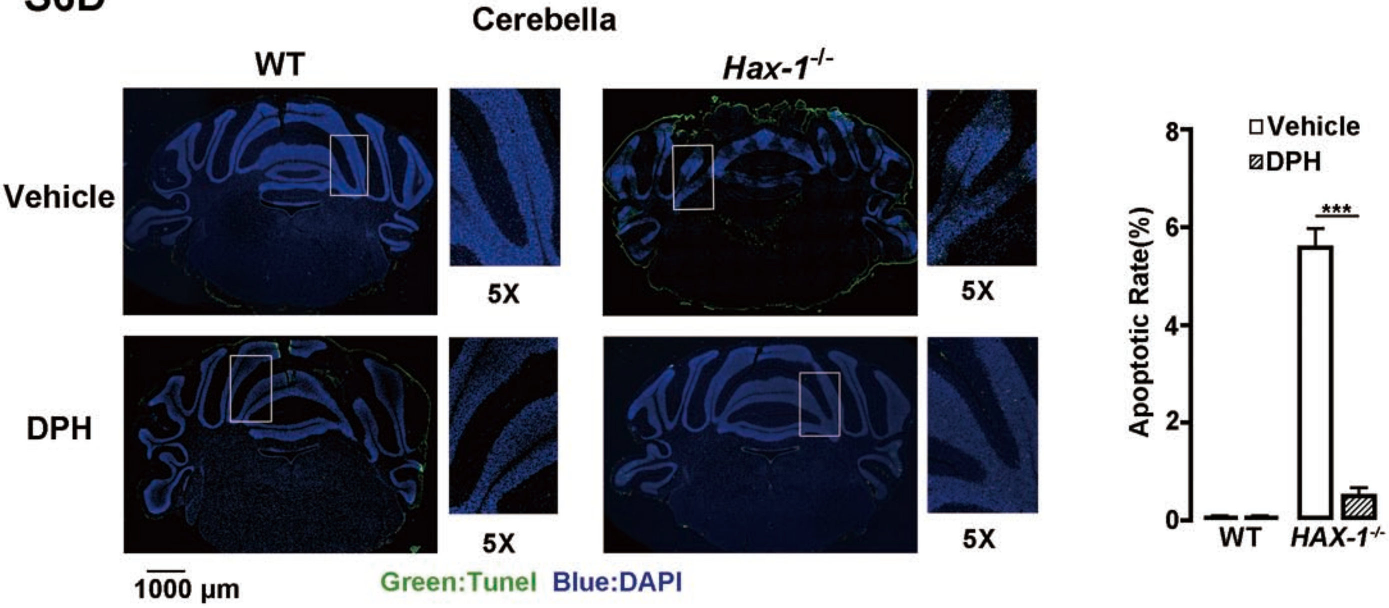

Supplementary Figure S6. c-Abl activation protected nerve cells from HAX-1 insufficiency-induced ROS accumulation and death. (S6A) The relative intensity of WB bands in Fig. 6A was quantified by gray scanning and represented as mean±S.D. of three independent analysis. ***p<0.001, Student's t test. Right panel, the Western blot analysis of cellular c-Abl levels normalized by beta-Actin in lysates of Fig. 6A. (S6B) MCF-7 cells were treated with 10 μM DPH for the indicated times, and cell lysates were immunoblotted with anti-p-Y207 or anti-CrKL antibody. (S6C) SH-SY5Y cells were transfected with PSR-GFP-siHAX-1 or PSR-GFP scramble plasmids, and HAX-1 levels were analyzed by Western blot (left). PMNs derived from BALB/C mouse spleens were transfected with lentivirus-HAX-1-siRNA or lentivirus-scramble-siRNA (MOI=5), and HAX-1-siRNA cells were cultured in 1640 medium for 96 h. Then, HAX-1 protein levels were analyzed (middle). The relative intensity of WB bands was quantified by gray scanning and represented as mean±S.D. of three independent analysis. ***p<0.001, Student's t test (right). (S6D) Cerebellum from Fig. 6E were subjected to terminal deoxynucleotidyl transferase-mediated dUTP nick end labeling (TUNEL) staining. TUNEL-positive cells are marked in green, and nuclei are marked with DAPI. Right panel, quantification and statistical analysis of TUNEL-positive cells. ***p<0.001, Student's t test.
