# Supplementary figures and images for: Anti-apoptotic HAX-1 suppresses cell apoptosis by promoting c-Abl kinase-involved ROS clearance
Source: Cell Death Dis. 2022 Apr 4;13(4):298. doi: 10.1038/s41419-022-04748-2 (PMC8979985; doi:10.1038/s41419-022-04748-2)

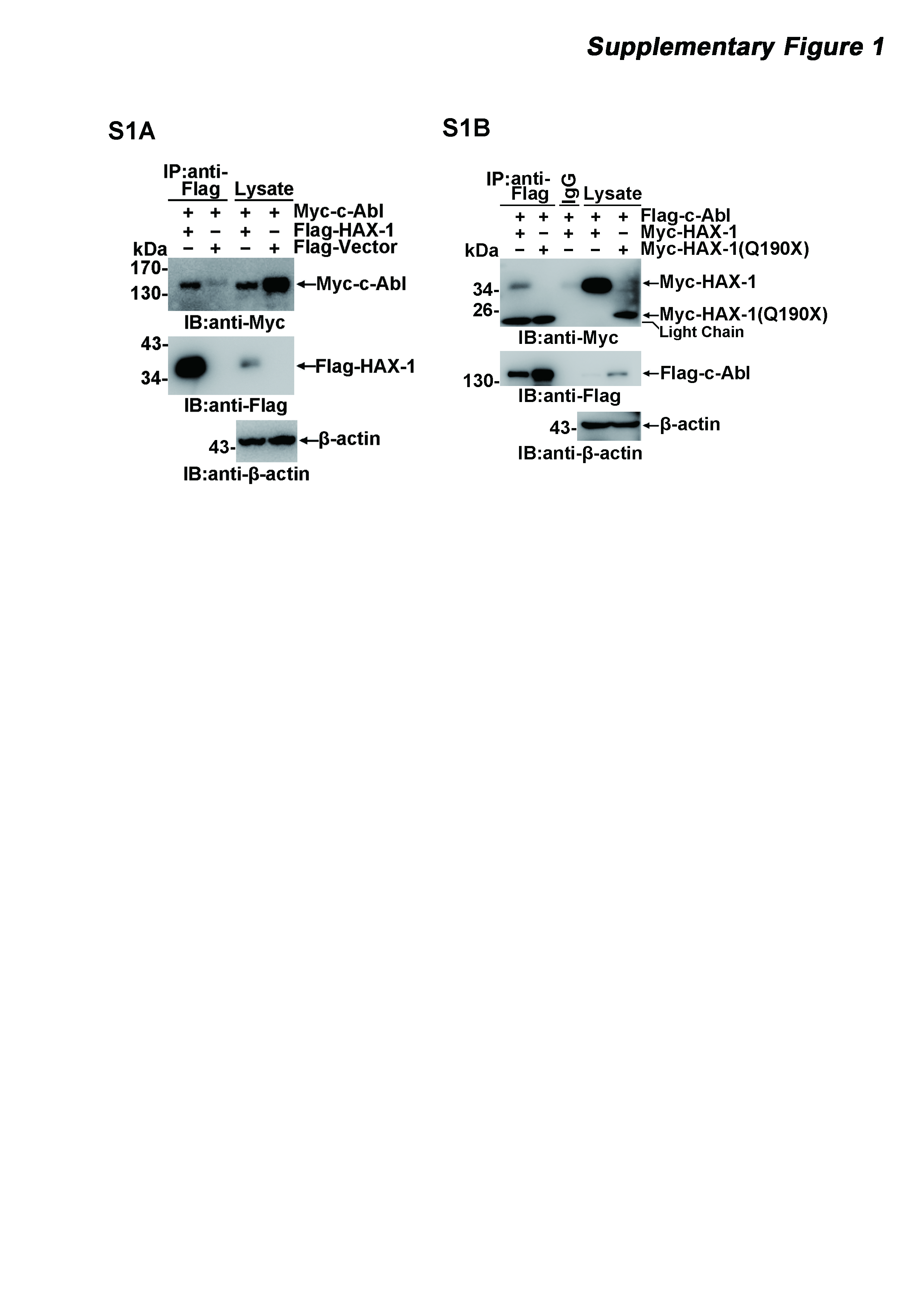

Supplement: Supplementary file 2 — Supplementary Figure 1 [file 41419_2022_4748_MOESM2_ESM.tif]

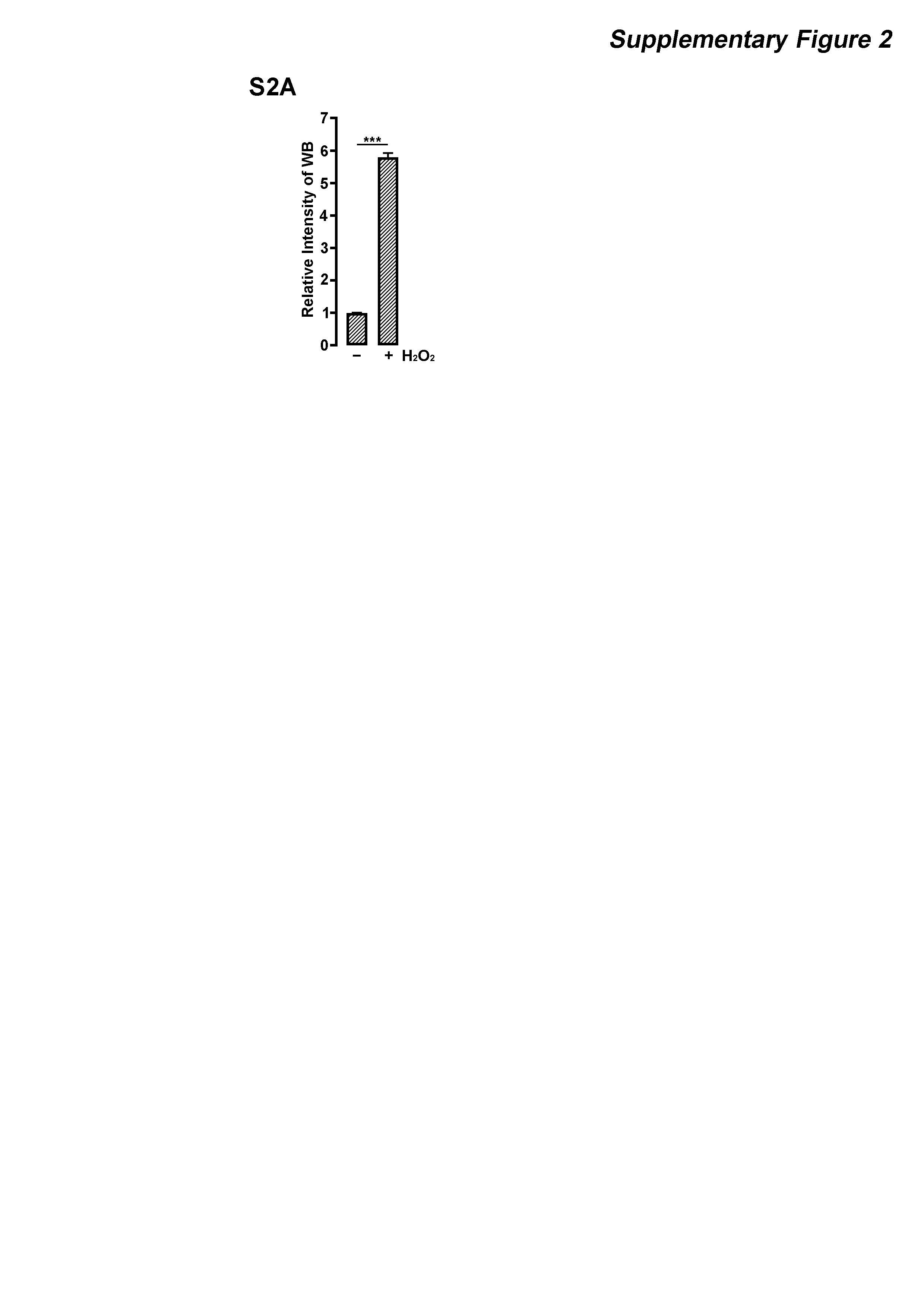

Supplement: Supplementary file 3 — Supplementary Figure 2 [file 41419_2022_4748_MOESM3_ESM.tif]

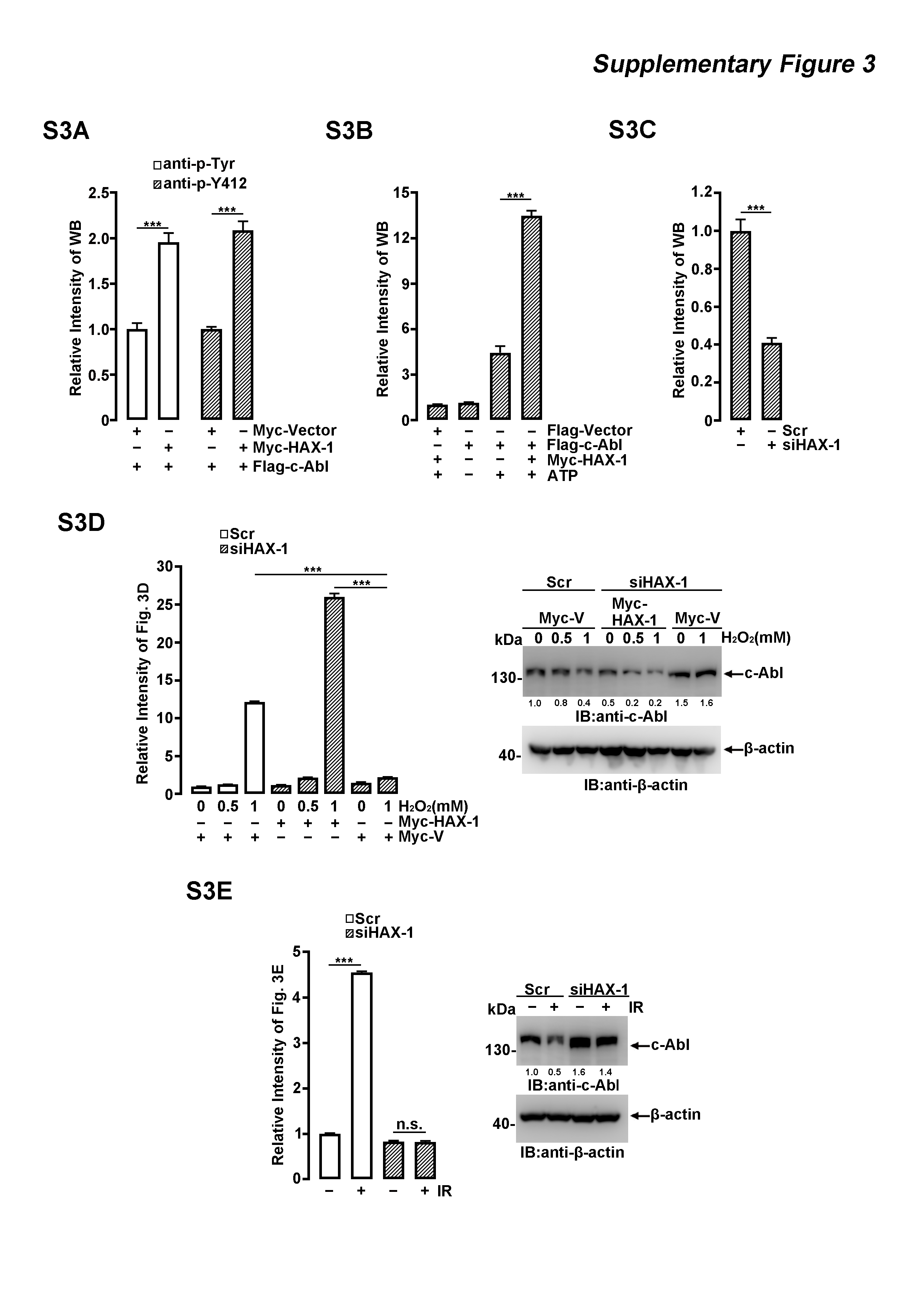

Supplement: Supplementary file 4 — Supplementary Figure 3A-E [file 41419_2022_4748_MOESM4_ESM.tif]

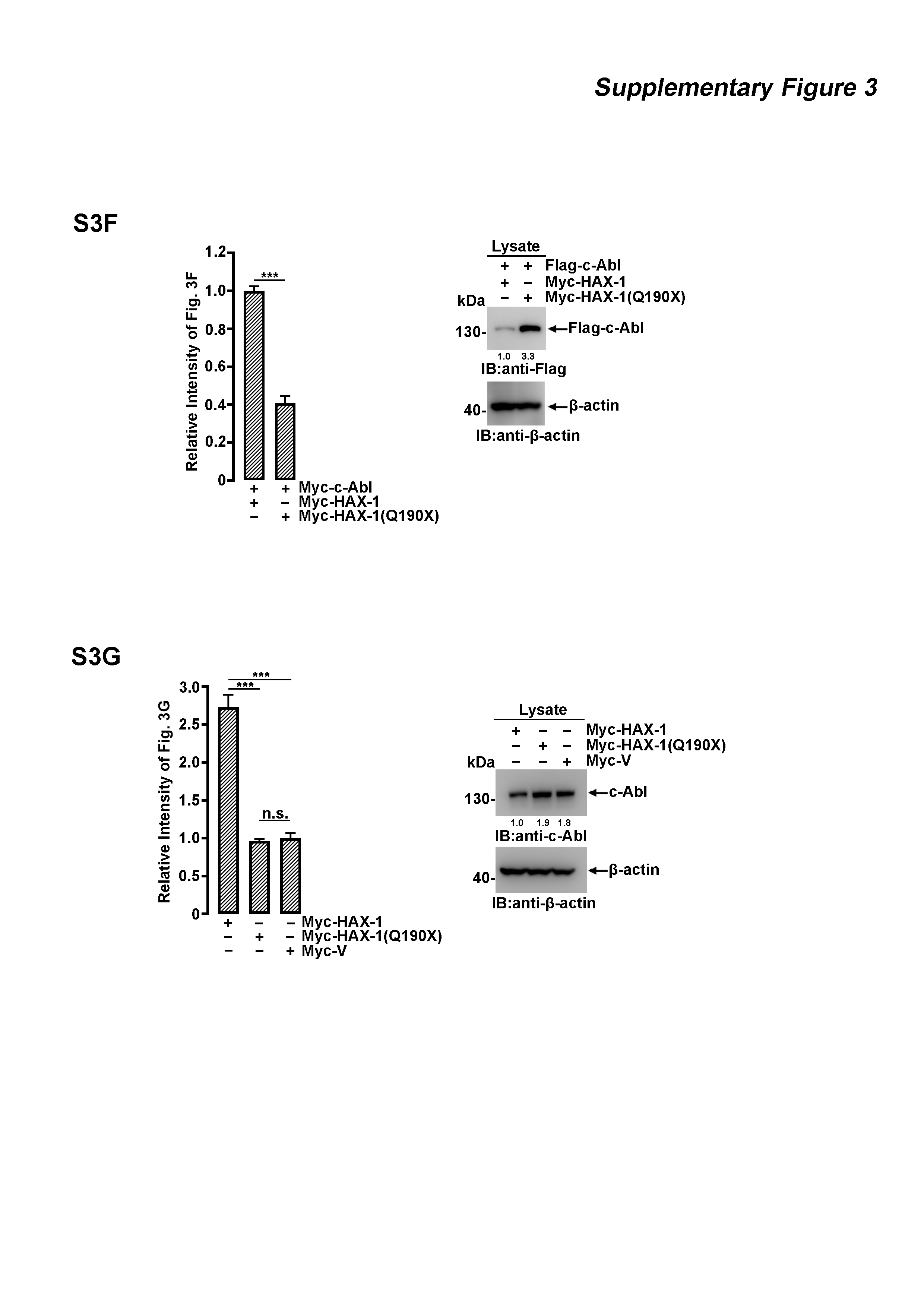

Supplement: Supplementary file 5 — Supplementary Figure 3F-G [file 41419_2022_4748_MOESM5_ESM.tif]

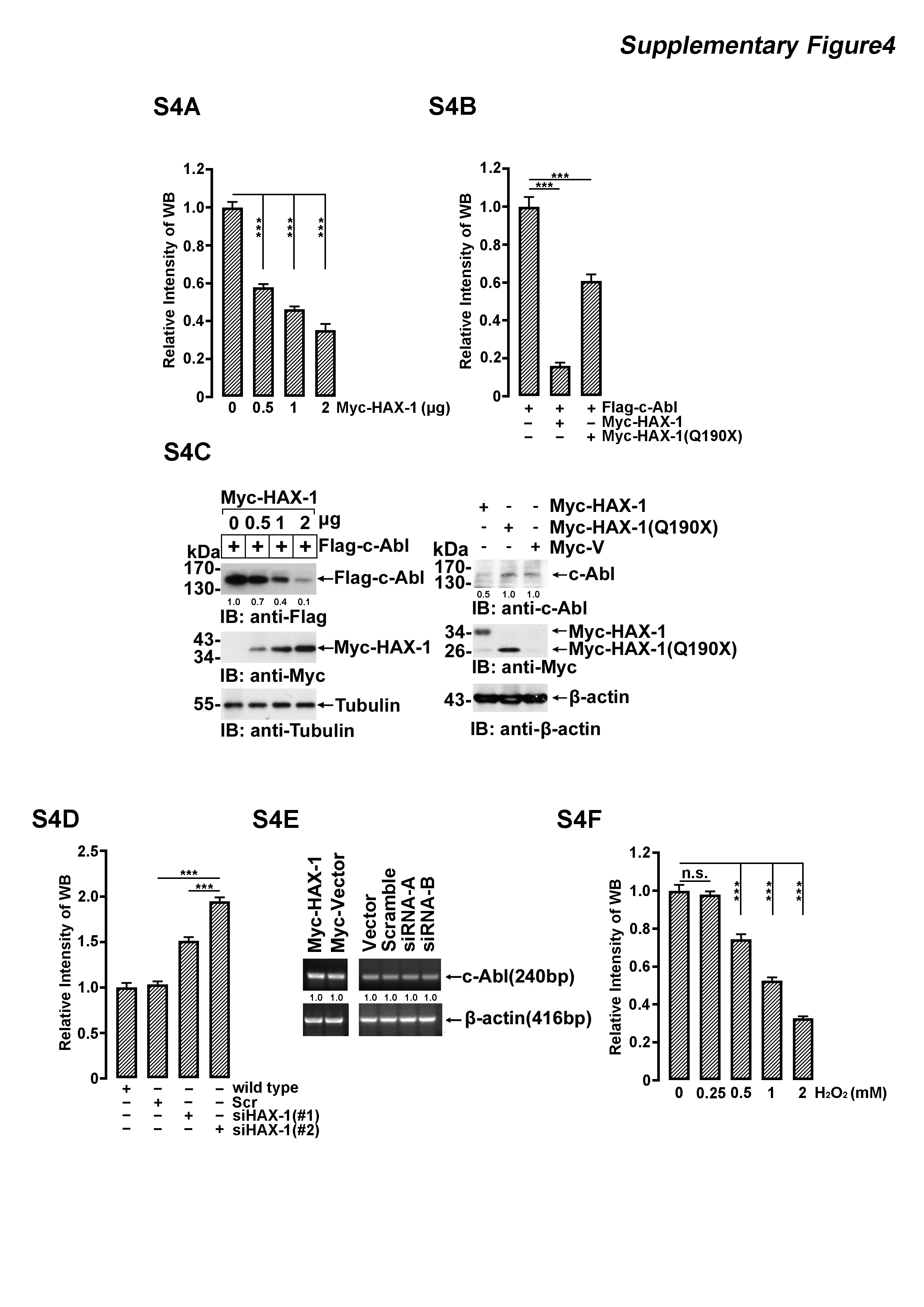

Supplement: Supplementary file 6 — Supplementary Figure 4A-F [file 41419_2022_4748_MOESM6_ESM.tif]

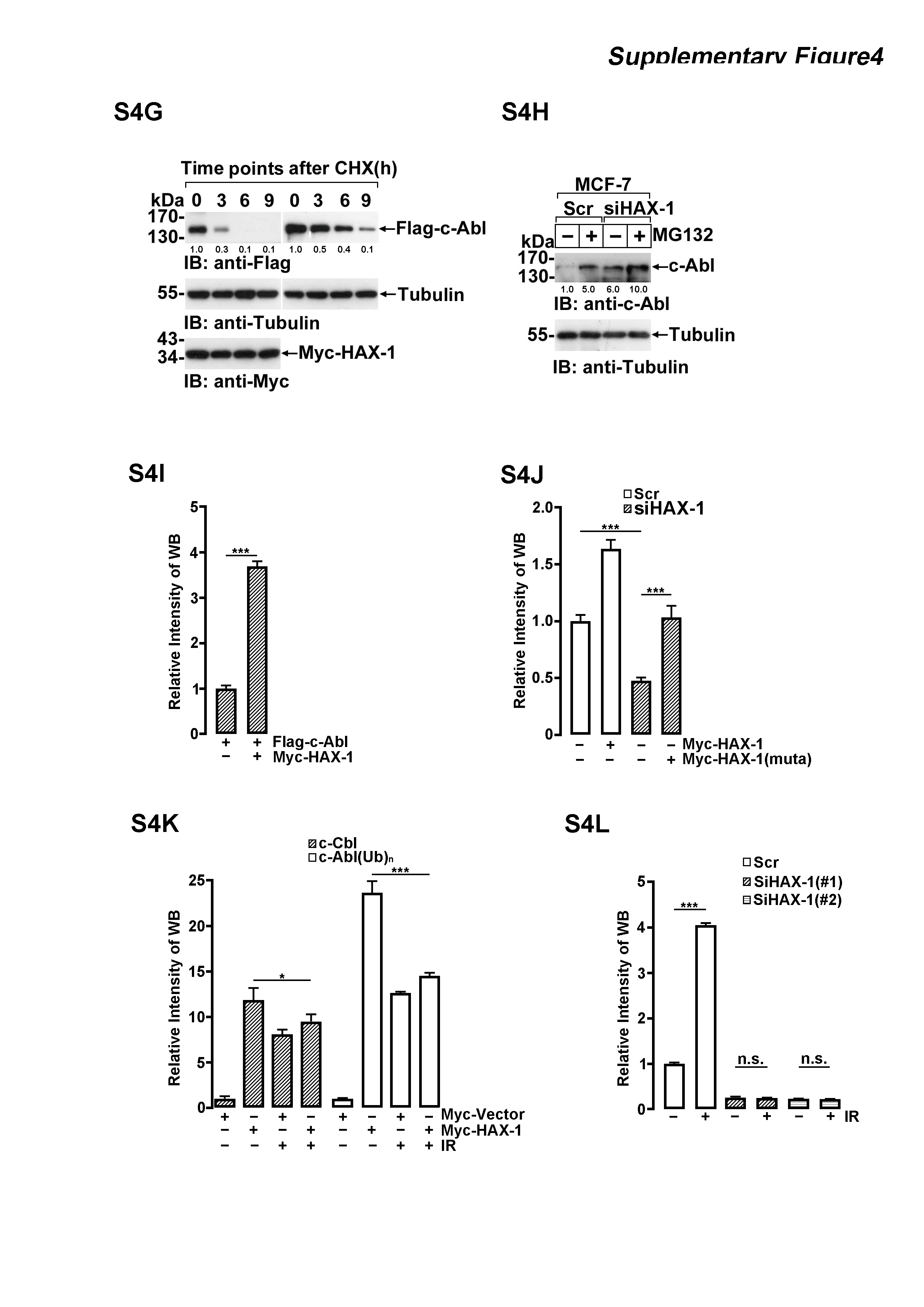

Supplement: Supplementary file 7 — Supplementary Figure 4G-L [file 41419_2022_4748_MOESM7_ESM.tif]

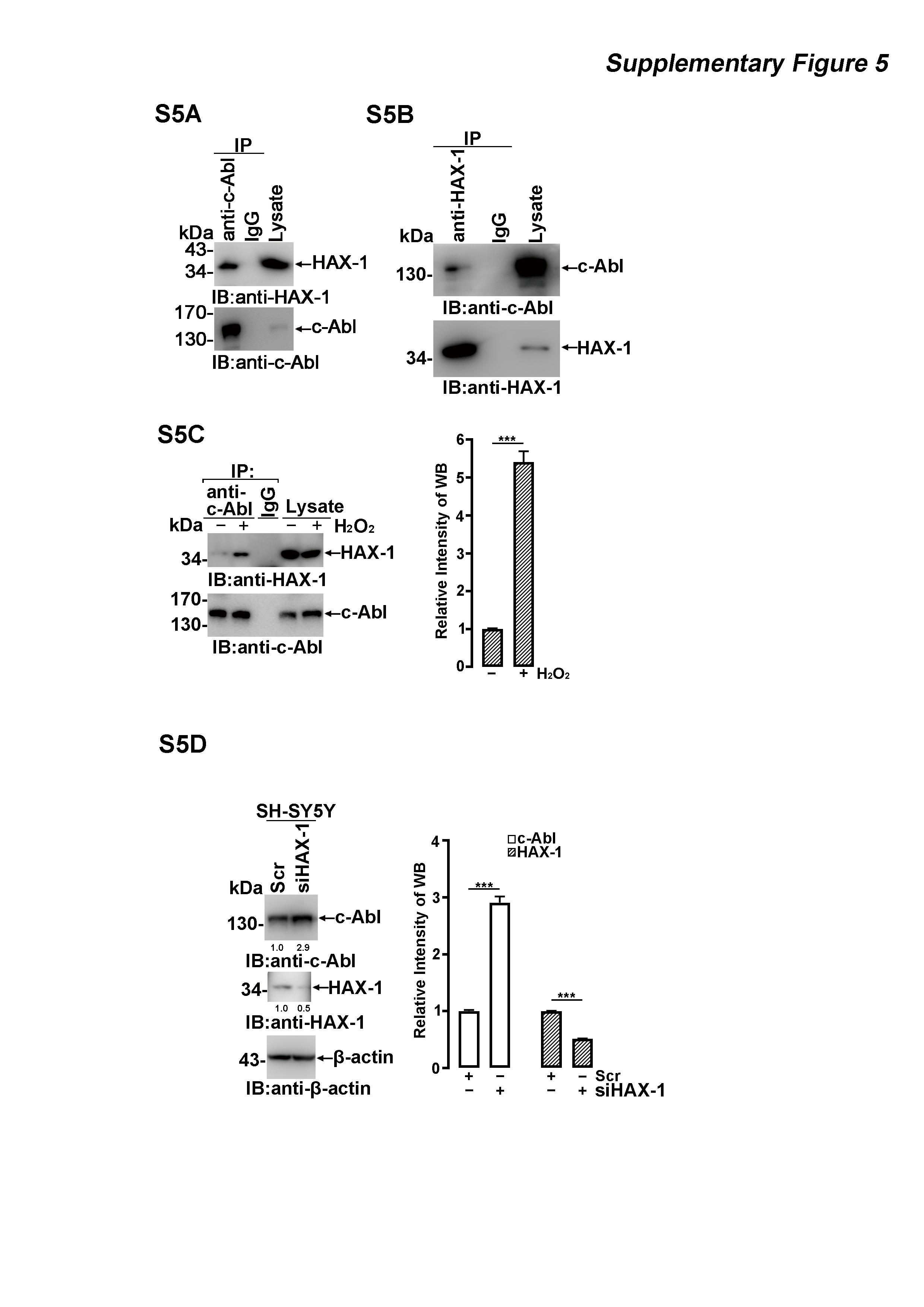

Supplement: Supplementary file 8 — Supplementary Figure 5 [file 41419_2022_4748_MOESM8_ESM.tif]

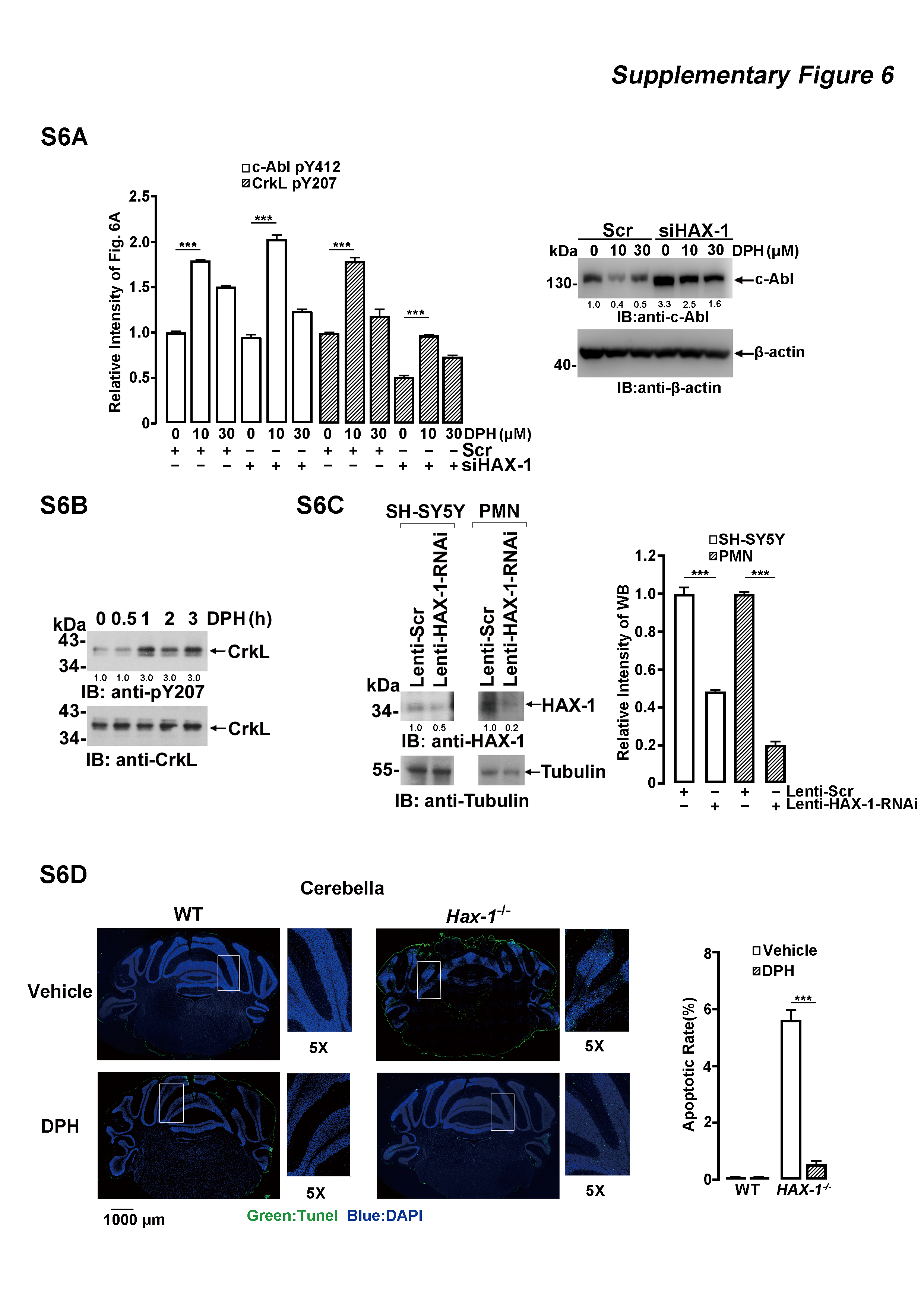

Supplement: Supplementary file 9 — Supplementary Figure 6 [file 41419_2022_4748_MOESM9_ESM.tif]
